# Explainable Multi-Layer Dynamic Ensemble Framework Optimized for Depression Detection and Severity Assessment

**DOI:** 10.3390/diagnostics14212385

**Published:** 2024-10-25

**Authors:** Dillan Imans, Tamer Abuhmed, Meshal Alharbi, Shaker El-Sappagh

**Affiliations:** 1 College of Computing and Informatics, Sungkyunkwan University, Suwon 16419, Republic of Korea; dillanimans@g.skku.edu (D.I.); shaker@skku.edu (S.E.-S.); 2 Department of Computer Science, College of Computer Engineering and Sciences, Prince Sattam Bin Abdulaziz University, Alkharj 11942, Saudi Arabia; mg.alharbi@psau.edu.sa; 3 Faculty of Computer Science and Engineering, Galala University, Suez 435611, Egypt; 4 Faculty of Computers and Artificial Intelligence, Benha University, Benha 13512, Egypt

**Keywords:** dynamic ensemble, explainable AI, depression detection, classifier optimization, machine learning

## Abstract

Background: Depression is a pervasive mental health condition, particularly affecting older adults, where early detection and intervention are essential to mitigate its impact. This study presents an explainable multi-layer dynamic ensemble framework designed to detect depression and assess its severity, aiming to improve diagnostic precision and provide insights into contributing health factors. Methods: Using data from the National Social Life, Health, and Aging Project (NSHAP), this framework combines classical machine learning models, static ensemble methods, and dynamic ensemble selection (DES) approaches across two stages: detection and severity prediction. The depression detection stage classifies individuals as normal or depressed, while the severity prediction stage further classifies depressed cases as mild or moderate-severe. Finally, a confirmation depression scale prediction model estimates depression severity scores to support the two stages. Explainable AI (XAI) techniques are applied to improve model interpretability, making the framework more suitable for clinical applications. Results: The framework’s FIRE-KNOP DES algorithm demonstrated high efficacy, achieving 88.33% accuracy in depression detection and 83.68% in severity prediction. XAI analysis identified mental and non-mental health indicators as significant factors in the framework’s performance, emphasizing the value of these features for accurate depression assessment. Conclusions: This study emphasizes the potential of dynamic ensemble learning in mental health assessments, particularly in detecting and evaluating depression severity. The findings provide a strong foundation for future use of dynamic ensemble frameworks in mental health assessments, demonstrating their potential for practical clinical applications.

## 1. Introduction

Major depressive disorder (MDD) is a pervasive and severe mental health condition that affects individuals globally, affecting all age groups, demographics, and socioeconomic boundaries. In 2023, more than 280 million people worldwide suffered from depression, making it one of the leading causes of mental disability [1]. In the United States, by 2020, the incremental economic burden of adults with MDD was around USD 326.2 billion, which includes direct costs, suicide-related costs, and workplace costs [2]. This substantial economic impact, along with the significant number of affected individuals, highlights the urgent need for early intervention strategies to alleviate the extensive personal, societal, and financial burdens of depression. The elderly population is particularly vulnerable to depression, experiencing significant impacts despite lower overall prevalence rates compared to younger age groups. In the United States, by 2021, approximately 4.5% of adults aged 50 years and older suffer from MDD [3]. This represents a substantial number of individuals who face depression, with possible significant under-reporting due to social stigma [4]. Factors such as chronic illness, loss of loved ones, and social isolation exacerbate depression in seniors. The impact on this age group is severe, leading to reduced quality of life, increased risk of physical illness, and higher mortality rates [5]. Furthermore, senior people with depression are more likely to experience functional impairments and a decrease in their ability to live independently, adding to the social and economic burden [5]. Early detection of depression is critical for several reasons. First, timely identification enables more effective treatment interventions [6]. Second, people with more severe depression often exhibit resistance to treatment, making early intervention crucial to prevent further deterioration [7,8,9,10]. Predicting the severity of depression is equally essential, as it allows for personalized care. Patients with mild depression can receive tailored interventions, while those with severe depression can be prioritized for more intensive treatment [11,12]. In addition, severe depression is strongly associated with an increased risk of suicide, making it vital to identify and prioritize these cases for urgent intervention [13]. Therefore, the ability to detect depression early and accurately predict its severity is essential for effective treatment and prevention strategies.

Currently, depression screening is heavily based on self-report tools such as the Patient Health Questionnaire 9 (PHQ-9), which is the most widely used screening instrument [14]. Although these tools have proven effective, they can be limited by factors such as delayed diagnosis due to individuals not being aware of their condition or unwilling to seek help due to stigma [4,15]. This can result in undiagnosed depression, and individuals can fall deeper into the disorder without timely intervention. Therefore, identifying additional factors that contribute to depression could help clinicians improve detection and severity prediction without relying solely on self-report measures.

In this study, we explore a promising approach to exploiting data from indirect self-reporting questionnaires on emotional health, social interactions, and daily experiences with machine learning (ML) to detect the presence of depression and predict the severity of depression. This approach also helps mitigate diagnosis delays, which often occur when individuals are either unaware of their condition or face barriers such as stigma or limited access to healthcare. Furthermore, self-report measures provide a noninvasive, private way for older adults to assess their mental health, encouraging more proactive engagement without the fear of judgment. Compared to clinical evaluations, indirect self-report measures are less expensive and more readily available, especially in community or telehealth settings. This approach promotes self-awareness and preventive care, empowering older adults to track their well-being and take timely action, whether through professional help or lifestyle changes.

A growing body of literature has explored the application of ML models to detect depression [16,17] and predict its severity [18,19]. However, a review of the existing literature reveals several limitations. Most studies mainly employ classical and static classifiers, which are limited in their adaptability and may not fully exploit the diversity of data [20,21]. Ensemble methods have proven their superior performance compared to classical methods [22,23,24,25]. However, static ensembles are based on a fixed pool of base classifiers, which might not be suitable for all test examples. As a result, the generalizability of these models is not good. The dynamic ensemble algorithms represent a more advanced approach, dynamically selecting and combining multiple models based on specific criteria for each instance or subset of instances [26,27]. Unlike static ensembles, which rely on a fixed set of models, dynamic ensembles adaptively choose the most appropriate prediction models, improving accuracy and performance [28]. This adaptability makes dynamic ensembles particularly suited for complex tasks such as depression detection. To our knowledge, no study in the literature has used dynamic ensembles to predict depression. Improving the model’s performance is insufficient to achieve a physician’s trustworthiness. There is a lack of personalized assessment in which the model can provide customized and tailored decisions for different individuals. In addition, model explainability is crucial in clinical settings to build trust and improve decision-making [29].

Many studies lack explainable artificial intelligence (XAI) techniques to understand how models detect depression or predict its severity, and they fail to identify the specific features or concepts that contribute to an individual’s depression. Our study aims to address these gaps by incorporating dynamic ensemble models and XAI techniques to provide a more comprehensive understanding of depression detection and severity prediction. In summary, the study contributes to the field with these points:

- We propose an explainable, multilayer framework for the detection and prediction of the severity of depression using dynamic ensemble selection (DES) techniques. The proposed framework comprises three distinct layers. The first layer functions as a detection model designed to predict the presence of depression. The second layer focuses on the severity prediction, predicting the degree of depression in patients already identified as depressed. Finally, the confirmation layer is a regression layer that estimates an individual’s PHQ-9 score based on their specific features. By integrating these three layers, our framework provides a comprehensive decision support framework that not only detects depression but also assesses its severity and validates the precision of the predictions through the estimation of the PHQ-9 score.
- In the detection and severity prediction phases, we assess the effectiveness of various ML models using the National Social Life, Health, and Aging Project (NSHAP) dataset, which comprises a diverse range of questionnaire responses from domains such as social networks and relationships, as well as biomarker data like saliva samples and blood pressure readings from older adults in the United States. This variety of features provides valuable insights into which factors most strongly influence depression. The models explored classical ML algorithms, static ensemble techniques, and, notably, a selection of DES methods. The inclusion of DES represents a novel approach, as it aims to assess whether DES can further optimize and enhance the performance of classical ML and static ensemble models.
- In the scale prediction layer, we evaluate the performance of various static ensemble regression models, alongside a voting regression model, in predicting the PHQ-9 score from the same dataset. This approach validates the results of both the detection and severity prediction layers, offering an additional confirmation of the accuracy and reliability of the overall predictions.
- We enhance the framework by incorporating XAI features to assist physicians in understanding the model’s decision-making processes and provide insight into what factors contribute to an individual’s depression presence or the severity of depression, especially among older adults. This is achieved by applying XAI techniques to the best-performing models in each of the three layers.

The study is organized as follows. Section 2 reviews the literature, Section 3 presents the proposed model, Section 4 describes the experimental setup, Section 5 describes the results of the three layers, Section 6 provides the model explainability and relevant factors contributing to depression, and finally, Section 7 concludes the study.

## 2. Related Work

This section offers an exhaustive review of recent advancements in the detection of depression, the prediction of severity of depression, and the prediction of depression scales using ML techniques. In addition, it examines the potential of dynamic ensemble methods, particularly within the mental health domain, highlighting studies that exhibit improved accuracy and adaptability in predictive modeling. The related work analyzed herein forms the basis for understanding the current research landscape and discerning the gaps that our study seeks to fill.

### 2.1. Depression Detection

The detection of depression involves evaluating individuals to determine the presence of MDD, often classified as normal or depressed. In clinical settings, standardized methods have been developed to diagnose depression, typically conducted in two primary ways. First, clinical interviews serve as a conventional approach in which a mental health professional evaluates an individual’s mood, behaviors, thoughts, and physical symptoms over a defined period (typically the last two weeks or more). Clinicians often rely on the Diagnostic and Statistical Manual of Mental Disorders, Fifth Edition (DSM-5), as a diagnostic checklist to identify depression symptoms. DSM-5 has shown significant reliability through test–retest studies, particularly in mood disorders. However, its reliability is relatively lower when diagnosing disorders such as MDD or generalized anxiety disorder compared to conditions such as autism spectrum disorder or borderline personality disorder [30]. In addition, numerous factors hinder people from seeking a clinical diagnosis, including societal stigma [4], financial barriers, perceived lack of emotional distress, and concerns about the efficacy of treatment [31]. Although clinical interviews remain the gold standard for diagnosis, associated barriers make large-scale depression detection difficult. As a result, self-report screening tools have emerged as a complementary solution.

Self-report screening instruments such as the PHQ-9 [32] and the Beck Depression Inventory (BDI) [33] are extensively used for depression detection. These instruments enable individuals to self-evaluate their mental health, providing a cost-effective and accessible alternative to clinical consultations. Empirical evidence demonstrates that these tools, particularly the PHQ-9, maintain reliability even in non-English-speaking countries [34,35]. Consequently, due to its established reliability, the PHQ-9 is employed as the principal metric in our research. Nonetheless, the self-administrative nature of these instruments necessitates that individuals actively seek evaluation and treatment, a task that can be challenging for those experiencing depressive symptoms [31]. In addition to how different cultures interpret and respond to PHQ-9 items, PHQ-9 alone can overdiagnose by generating false positives, particularly in patients with conditions such as bipolar disorder, anxiety, or other psychiatric disorders [36]. Although PHQ-9 serves as a valuable instrument, it is recommended that its results be utilized alongside other robust diagnostic assessment tools to achieve a more comprehensive evaluation of depression, including severe cases, and avoid false positives [37,38]. This study integrates ML techniques applied to multiple diagnostic and clinical assessments to proactively identify individuals at risk, thereby avoiding exclusive dependence on items from PHQ-9.

In recent years, numerous studies have explored the application of ML techniques to detect depression. These approaches span various modalities, from audiovisual data to neurophysiological responses and social media activity, each aimed at improving the precision and timeliness of depression detection [39,40]. For example, Min et al. [16] leveraged audiovisual features from YouTube videos, demonstrating the potential of combining audio and visual data for detecting depressive behaviors. Their XGBoost model achieved a 75.85% accuracy, highlighting that audiovisual features were particularly effective in early detection on social media platforms. However, while this approach underscores the value of integrating YouTube videos in mental health research, it is limited by the dependence on user-generated content, which may lack consistency and introduce noise into the data. In contrast, Li et al. [17] focused on a more controlled setting, using neurophysiological data as electrophysiological responses (ERP) during a dot-probe task to study attentional bias in patients with MDD. Their study used correlated feature selection to enhance the classification accuracy, achieving a high rate of 94% with the K-Nearest Neighbor classifier. This method of using ERP data, particularly the P300 component, provided more reliable signals directly linked to brain activity. However, despite the high accuracy, ERP-based methods require specialized equipment, making them less scalable and more resource-intensive compared to audiovisual data analysis. A different modality, social media texts, was explored by Govindasamy and Palanichamy [41], who applied sentiment analysis on Twitter data to detect depression using Naive Bayes and hybrid Naive Bayes Decision Tree classifiers. Their study achieved accuracies of 92.34% and 97.31% on datasets of 1000 and 3000 tweets, respectively. This approach benefits from the large volume of publicly available social media data, allowing for scalable depression detection. However, sentiment analysis is highly dependent on the quality of the labeled data, and the models may struggle with nuances in language or cultural differences in expressing emotions, which could affect their generalizability across different populations. Malik, Shabaz, and Asenso [42] took yet another approach by applying ML techniques to survey-based data, combining Decision Tree, K-Nearest Neighbor, and Naive Bayes classifiers to analyze responses from 1694 individuals. Their study found the K-Nearest Neighbor to be the most effective, with an accuracy of 92.32%. This method, relying on structured survey responses, provided an accessible way to detect depression. However, surveys might not fully capture the dynamic nature of depressive symptoms, unlike continuous monitoring via physiological data. When comparing these traditional ML approaches, it is evident that each study offers specific strengths but also faces unique limitations based on the type of data used. For instance, models relying on structured data like surveys or sociodemographic factors may offer simplicity and accessibility but risk missing out on the complexity of depressive behaviors, which could be captured better through neurophysiological, behavioral data, or health exam data. On the other hand, studies using neurophysiological signals or audiovisual data offer greater accuracy but at the cost of requiring more specialized equipment or large-scale user engagement.

Recent advances in deep learning have also been applied to depression detection, with more sophisticated results but new challenges. Acharya et al. [43] employed a convolutional neural network (CNN) model to analyze electroencephalogram (EEG) data, achieving accuracies of 93.5% and 96.0% using signals from the left and right hemispheres, respectively. Their study supports the hypothesis that depression is linked to hyperactivity in the right hemisphere. While EEG-based deep learning models perform well in detecting depression, their application is limited by the need for specialized equipment and the complexity of interpreting EEG data, especially in large-scale deployments. Marriwala and Chaudhar [44] introduced a hybrid deep learning model combining textual and audio features, applying Long Short Term Memory (LSTM) and Bidirectional LSTM (Bi-LSTM) models to the DAIC-WoZ database. Their findings showed that the audio CNN model outperformed the textual model, achieving an impressive accuracy of 98%, highlighting the effectiveness of audio features in detecting depression. However, like other deep learning models, these approaches require extensive computational resources and large datasets to generalize well. This poses a challenge for smaller-scale datasets like the 2005–2006 NSHAP dataset used in this research, which lacks the volume and diversity necessary for training deep learning models effectively. Moreover, while deep learning models demonstrate higher accuracy, they often operate as “black boxes”, making it difficult to interpret the results or understand the importance of individual features. In contrast, classical ML models not only perform comparably well with fewer resources but also offer easier integration with XAI techniques, providing clearer insights into feature importance and model decision-making processes.

### 2.2. Depression Severity Prediction

Depression severity prediction involves determining the level of depression in an individual, which is critical to providing appropriate care and intervention. Various screening tools categorize severity differently. For example, PHQ-9 divides depression into categories such as mild, moderate, moderately severe, and severe [32], while the BDI uses minimal, mild, moderate, and severe [33]. Self-screening tools such as these are more commonly used to categorize the severity of depression than DSM-5 since DSM-5 is typically used to diagnose depression as present or absent. Although similar to depression detection, prediction of severity faces unique challenges. A significant limitation of self-screening tools is that depressed individuals may be reluctant to seek self-evaluation [31], thus preventing clinical evaluation and accurate severity assessment. This issue is particularly concerning for those with severe depression, as they have a higher risk of suicide and other comorbidities [13]. Therefore, accurate prediction of the severity of depression, especially after an initial diagnosis of depression, is essential for appropriate and timely intervention. Here, ML techniques can play a crucial role in enhancing the accuracy and efficiency of severity prediction.

Many studies have explored the application of ML to predict the severity of depression, often using diverse data sources such as biomarkers, functional brain activity, and behavioral data. These studies vary significantly in their approaches, each presenting unique strengths and limitations. Bader et al. [45] explored the combination of oxidative stress biomarkers (e.g., 8-isoprostane, 8-OHdG, and glutathione) with sociodemographic and clinical data to predict depression severity using ML models. The study demonstrated that integrating biomarkers with additional health-related factors improved detection accuracy, with oxidative stress markers ranked as the most critical predictors. While the Random Forest classifier consistently outperformed other models, the limitation here lies in the complexity of collecting and analyzing biomarkers, which may not be readily accessible in all healthcare settings. Furthermore, biomarkers like oxidative stress markers may vary based on factors unrelated to depression, such as physical health conditions, which could introduce noise into the model and limit its generalizability. In contrast, Huang et al. [46] took a functional near-infrared spectroscopy (fNIRS) approach, analyzing brain activity to classify mild and severe depression. Their support vector machine model achieved a high accuracy of 92.8%, demonstrating the effectiveness of combining temporal and correlation features from brain data. While this method offers a more objective diagnostic tool, the primary limitation is its dependency on specialized equipment (fNIRS), which is costly and requires technical expertise, making it less feasible for widespread clinical use. Additionally, like oxidative biomarkers, brain activity data may reflect other cognitive or physiological states, potentially affecting the model’s specificity in real-world applications. Choudhary et al. [47] took a different approach by leveraging passive smartphone data, such as digital behavioral markers and gyroscope sensor data, to predict depression severity. This method offers a non-invasive and continuous monitoring solution, achieving an accuracy of 87% for a two-class model (none vs. severe) and 78% for a three-class model. However, while smartphone data are scalable and convenient, its reliance on self-reported PHQ-9 scores as a ground truth introduces subjectivity, and the slight reduction in accuracy when incorporating gyroscope data suggests potential noise from irrelevant data sources. Shin et al. [48] examined voice as a potential biomarker for detecting both minor and major depressive episodes, utilizing voice features extracted from interviews. Their model achieved an Area Under Curve (AUC) of 65.9%, with 65.6% sensitivity and 66.2% specificity, indicating the potential for voice analysis in distinguishing between depression severities. However, a major limitation of this study is the small sample size (93 participants), which hinders the generalizability of the results. Moreover, voice data can be influenced by numerous external factors, such as physical illness or environmental noise, which complicates its use as a standalone biomarker for depression severity. Additionally, the relatively low performance of the model compared to others in the field suggests that more research is needed to fully capture the relationship between voice characteristics and depression severity.

Across these studies, the contrast between objective physiological data (biomarkers, brain activity, and voice features) and passive behavioral data (smartphone usage) is clear. While physiological data often lead to higher predictive accuracy, the need for specialized equipment (e.g., fNIRS, oxidative stress biomarker assays) limits the scalability and accessibility of these approaches. On the other hand, behavioral data collected from smartphones provides a scalable, non-invasive solution but is prone to noise and subjectivity, especially when paired with self-reported depression scores.

In addition to traditional ML methods, deep learning techniques have been applied to predict depression severity, demonstrating high accuracy but also introducing new challenges. Mao et al. [49] employed a multimodal approach, combining speech and text data to predict depression severity across five classes. Their model, trained on the DAIC-WOZ dataset, achieved an impressive F1-score of 0.9870 at the sequence level and 0.9074 at the patient level for the audio modality. Despite the promising results, the approach faces significant limitations due to the resource-intensive nature of deep learning models. These models require large datasets, high computational power, and often function as “black boxes”, making it difficult to interpret feature importance and understand how predictions are made. This lack of transparency and the computational demands pose challenges for their practical deployment in clinical settings.

When comparing deep learning approaches to traditional ML models, the contrast between performance and explainability becomes apparent. While deep learning models like those proposed by Mao et al. achieve higher accuracy in predicting depression severity, they suffer from black-box limitations and require substantial data and computational resources. In contrast, traditional ML models, while often slightly less accurate, provide more interpretable results and can be applied in settings where computational power or large datasets are not readily available. The trade-off between interpretability and performance is a key consideration in selecting the appropriate model for predicting depression severity.

### 2.3. Depression Scale Prediction

Depression scales, such as the PHQ-9, are widely used due to their ease of use, low cost, and ability to provide quantitative estimates for both depression diagnosis and severity. Our study used the PHQ-9 scale, similar to many of the studies mentioned. Although these studies may use different versions of the PHQ-9, such as the PHQ-8 or variations adapted for specific populations, they still offer valuable validation for using a regression layer in predicting depression severity. This alignment strengthens the methodological consistency of our model, ensuring that our numerical estimation approach is grounded in well-established clinical practice.

Jin et al. [50] developed a generalized multilevel Poisson regression model to predict depression severity in patients with diabetes, using PHQ-9 scores to assess depression over time. With 29 factors analyzed and a root mean square error (RMSE) of around 4, their model provided both population-level and patient-specific predictions. Although Jin et al. focused on diabetes, limiting the generalizability to broader populations, their use of longitudinal data and PHQ-9 reinforces the validity of our approach. However, the reliance on clinical trial data, which may not reflect real-world conditions, introduces a potential limitation. Syed et al. [51] used a different version of the PHQ-9 scale as part of the audio/visual emotion challenge to predict depression severity based on biomarkers of psychomotor retardation, including audio, video, and motion capture data. Their model achieved an RMSE of 6.34, indicating more errors compared to Jin et al.’s study. The higher error, alongside the complexity of data collection using motion capture, suggests that while multimodal data offer comprehensive insights, it also introduces noise and challenges in practical application. Aharonson et al. [52] used PHQ-8 scores to predict depression severity from speech data, introducing two ML architectures. Their second model, which grouped participants by severity class before applying regression, achieved an RMSE of 4.1, outperforming previous studies with RMSEs between 6.32 and 6.94. However, a limitation of Aharonson et al.’s work is the small dataset size (189 participants), which limits generalizability. Additionally, while speech-based models are scalable, they can suffer from variability in audio quality, potentially reducing prediction accuracy in diverse environments.

Despite the limitations presented in these studies—such as the focus on specific populations in Jin et al.’s study [50], the complexity of multimodal data collection in Syed et al.’s work [51], and the small dataset size in Aharonson et al.’s research [52]—they collectively demonstrate that using the PHQ-9 scale for predicting depression severity is a valid and reliable approach. These studies validate the PHQ-9 and its variants (e.g., PHQ-8) as effective tools in ML models for quantifying depression levels, offering robust performance across various methodologies and data sources. The consistency of results, despite different contexts and datasets, further supports the applicability of the PHQ-9 scale in predictive models for depression diagnosis and severity assessment, underscoring its value in both clinical and ML environments.

### 2.4. Dynamic Ensemble

Model stability and performance can be significantly improved through the use of ensemble methods, which include techniques like bagging, boosting, and stacking [53,54,55]. These methods typically employ a variety of base models, ranging from decision trees to more advanced classifiers such as support vector machines and neural networks. Recent studies have successfully applied these ensemble techniques to tasks like depression detection and severity prediction, utilizing classifiers such as random forests, gradient-boosting machines, and deep learning models, as discussed earlier. However, most of these studies have relied on static ensemble techniques, where the selection of base classifiers occurs only once during the training phase. A novel and increasingly promising approach to ensemble learning is DES, in which classifiers are dynamically chosen for each new instance to be classified [28]. In DES, the system first evaluates the competence level of each classifier in a pool of available classifiers. Based on this assessment, an ensemble is dynamically formed, selecting the most competent classifiers to predict the label for the specific query sample. The key idea behind DES is that not every classifier is equally capable of classifying all unknown samples; instead, each classifier specializes in a distinct local region of the feature space. Therefore, the challenge lies in dynamically identifying and selecting the most suitable classifiers for each individual sample. Various methods have been developed to address this, including techniques such as KNORA-E, KNORA-U, DES-KNN, and the Frienemy Indecision Region (FIRE) framework [56]. Several studies have explored the use of dynamic ensemble methods in medical applications. For example, KP, Muhammed Niyas, and Thiyagarajan (2021) aimed to improve the classification of healthy individuals, patients with mild cognitive impairment (MCI) and patients with Alzheimer’s disease (AD) at the baseline stage using data from the Alzheimer’s Disease Neuroimaging Initiative-TADPOLE dataset. This dataset includes multimodal features such as medical imaging, cerebrospinal fluid, cognitive tests, and demographic information. The study compared the performance of DES algorithms with traditional ML classifiers, evaluating both based on metrics like balanced classification accuracy, sensitivity, and specificity. Their results showed that the DES algorithms improved the classifier performance, particularly in distinguishing between healthy individuals, patients with MCI, and those with AD [57]. In the context of depression, Janardhan, Naulegari, and Nandhini Kumaresh [58] developed a four-stage ML classification system for the detection of depression using acoustic parameters. Speech recordings were obtained from the DAIC-WOZ dataset, and the eGeMAPS feature set was extracted. To address the class imbalance, adaptive synthetic resampling and data preprocessing were applied. Three feature selection methods were used: Borruta, SVM-RFE, and Fisher score to identify relevant features. In the fourth stage, several classifiers were tested with hyperparameter tuning performed via GridSearchCV during a 10-fold cross-validation. DES classifiers, particularly the KNORAU, were used to improve accuracy. The study found that the KNORAU, using 15 features selected by Fisher’s score, achieved the highest accuracy compared to individual classical ML or static ensemble classifiers on the DAIC-WOZ dataset. Despite the potential effectiveness of dynamic ensemble methods, very few studies in the literature have applied them to depression analysis. To our knowledge, no existing studies have yet utilized dynamic ensemble techniques in a unified framework for both depression detection and severity prediction. This represents a significant gap in current research, highlighting the potential of future work to explore the benefits of dynamic ensemble methods in comprehensive depression analysis.

## 3. Proposed Framework

**Figure 1:**
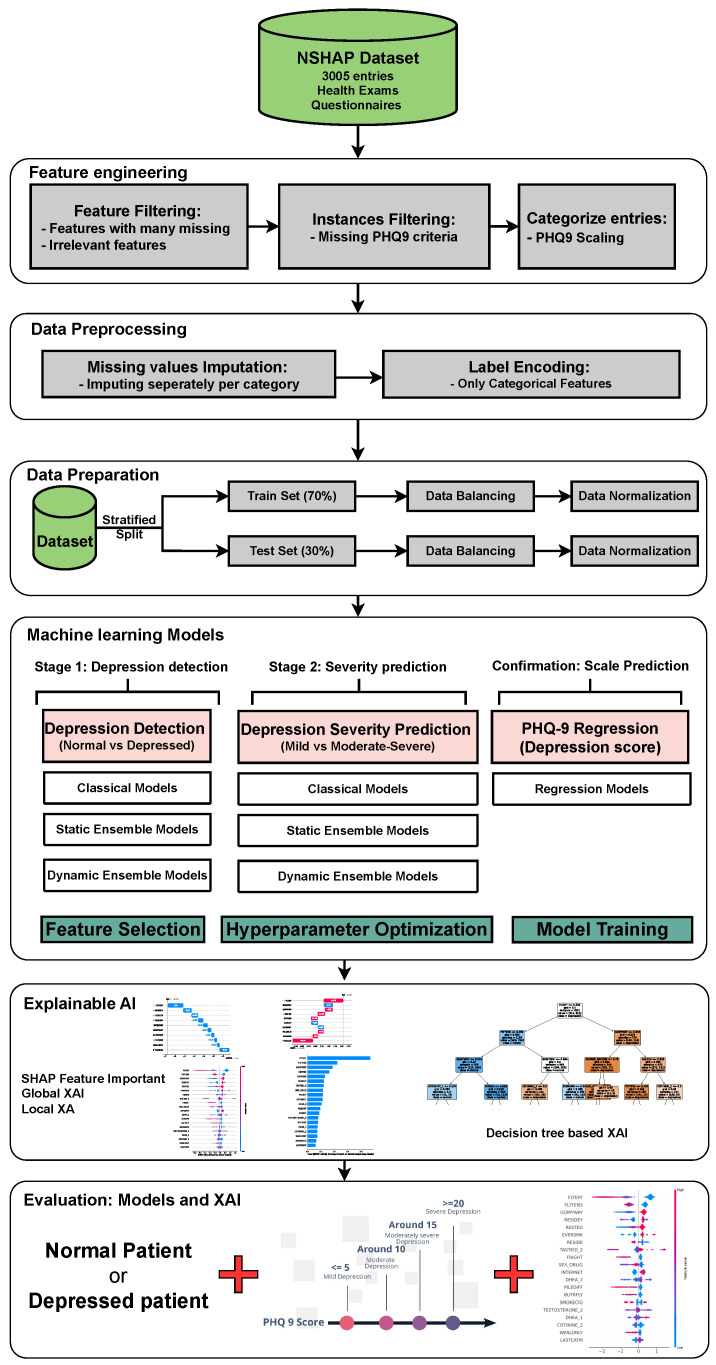
The architecture of the proposed framework. Abbreviations: National Social Life, Health, and Aging Project (NSHAP); explainable artificial intelligence (XAI).

Figure 1 illustrates the proposed model for depression detection, severity prediction, and scale prediction. The primary objective of this model is to deliver accurate and interpretable predictions. The model is structured into two main layers and one confirmation layer. The two main layers include the detection layer, which distinguishes between individuals with MDD and individuals without MDD, as well as the severity prediction layer, which differentiates between mildly depressed and moderately–severely depressed individuals. The third layer, referred to as the “confirmation layer” serves as a regression layer that predicts the PHQ-9 score for each individual. This layer further substantiates the predictions made by the two main layers, providing an additional level of validation and reinforcing the accuracy and reliability of the overall model. Each of the two main layers will utilize a collection of ML models, including classical ML models, static ensemble models, and DES models. The confirmation layer will only use static ensemble regressors. These models are compared using default hyperparameters with no feature selection, default hyperparameters with feature selection, and optimized hyperparameters with feature selection. The model with the best statistical performance is then extended to add XAI capabilities using different techniques. For ML engineers, XAI helps evaluate model stability, identify biases, and ensure that decisions are based on sound, interpretable logic, thereby improving model performance and trustworthiness. In clinical settings, XAI enables healthcare professionals to better understand the key factors driving model predictions, improving confidence in the model’s decision, and facilitating more informed diagnoses and treatments for MDD. This fosters greater trust between clinicians and AI systems, promoting responsible deployment of AI in mental health care.

### 3.1. Data Collection

This study uses the 2005–2006 iteration of the NSHAP dataset. Initiated in 2005–2006, NSHAP is a comprehensive longitudinal study aimed at examining the intricate relationships between social connections, health outcomes, and aging among older adults in the United States. Conducted by the National Opinion Research Center in collaboration with principal investigators from the University of Chicago, the study involved more than 3000 face-to-face interviews and the collection of biomeasures within participants’ homes. The sample was nationally representative, consisting of adults aged 57 to 85. The NSHAP dataset offers a wealth of variables that span multiple domains, including social networks, relationships, interviews, physical health, and biomarker data, such as saliva samples and blood pressure readings. In addition, the data set includes a robust set of mental health assessments, providing valuable information on the psychological well-being of the participants. These mental health measures are especially pertinent to study the prevalence and impact of conditions such as depression in the aging population [59]. This dataset is well suited for our research objectives due to its extensive variety of features, which allow for a comprehensive analysis of the factors that contribute to depression. Not only does it allow us to identify key contributors to depression in individuals, but it also provides the opportunity to explore the factors that exacerbate more severe forms of depression. These data are available at https://doi.org/10.3886/ICPSR20541.v10 (accessed on 25 April 2024).

### 3.2. Feature and Entry Exclusion

#### 3.2.1. Feature Exclusion

**Figure 2:**
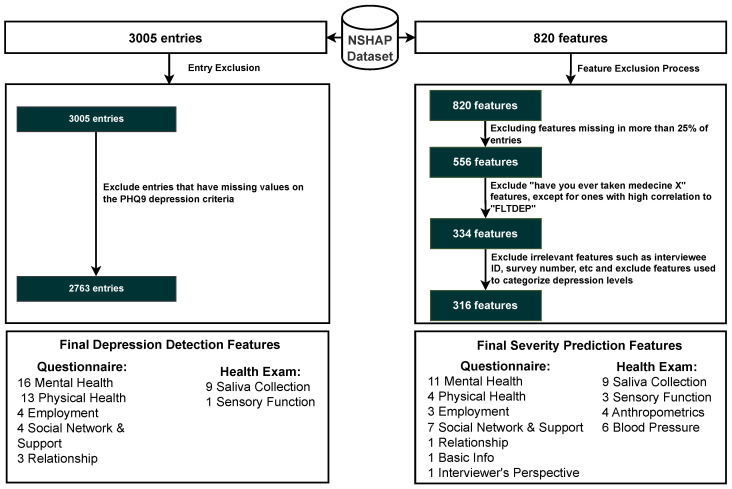
Inclusion criteria for entry and features. Abbreviations: Patient Health Questionnaire 9 (PHQ-9).

The NSHAP dataset originally comprised 820 features, but only 316 features were selected based on specific criteria. Initially, 264 features were excluded because they were missing in more than 25% of the entries. Subsequently, 222 features related to questions about whether participants had ever taken specific medications were removed, as the majority of responses were negative, indicating low relevance. However, specific medication features that showed a high correlation with the FLTDEP variable (felt depression) were retained to identify possible connections to depression. In addition, nine irrelevant features, such as interview IDs or survey numbers, that do not have analytical value were removed. The other nine features directly used to categorize depression levels were also removed to avoid training the model on the same criteria used for categorization, ensuring unbiased model development. By excluding features with a high percentage of missing data and those with low relevance, the model becomes more robust to noise and better equipped to generalize to new data. The elimination of irrelevant or redundant features reduces the risk of overfitting, where the model might learn specific patterns that do not generalize beyond the training dataset. Furthermore, focusing on the most relevant features allows the model to learn meaningful relationships that contribute to better predictions of depression. The inclusion of medication features with high correlation to depression ensures that the model remains sensitive to factors that might influence mental health, improving its ability to identify at-risk individuals. Overall, this careful feature selection enhances the model’s accuracy, interpretability, and efficiency in both training and deployment scenarios. This selection process resulted in 316 features, which are organized into categories, as shown in Figure 2. These categories are divided by the original researchers of this dataset into questionnaire features and health examination features, each with their subcategories, which facilitates a structured approach to the analysis in Section 6 [59].

#### 3.2.2. Entry Exclusion

The initial NSHAP dataset consisted of 3005 individuals. However, due to some entries containing missing responses to the questions used to calculate PHQ-9 scores, only 2763 entries were deemed suitable for analysis. Although imputation techniques can be applied to other variables within the dataset, imputing values for the PHQ-9 was deemed inappropriate, as this instrument serves as the primary measure for categorizing depression status. Excluding entries with missing PHQ-9 responses ensures that the dataset used for model training and testing is reliable and free from the biases that could arise from imputed values for this variable. Of the remaining 2763 entries, 1308 individuals were classified as having no depression symptoms (normal), while 1455 were classified as depressed.

### 3.3. Classifying Entries Through the PHQ-9 Scale

**Figure 3:**
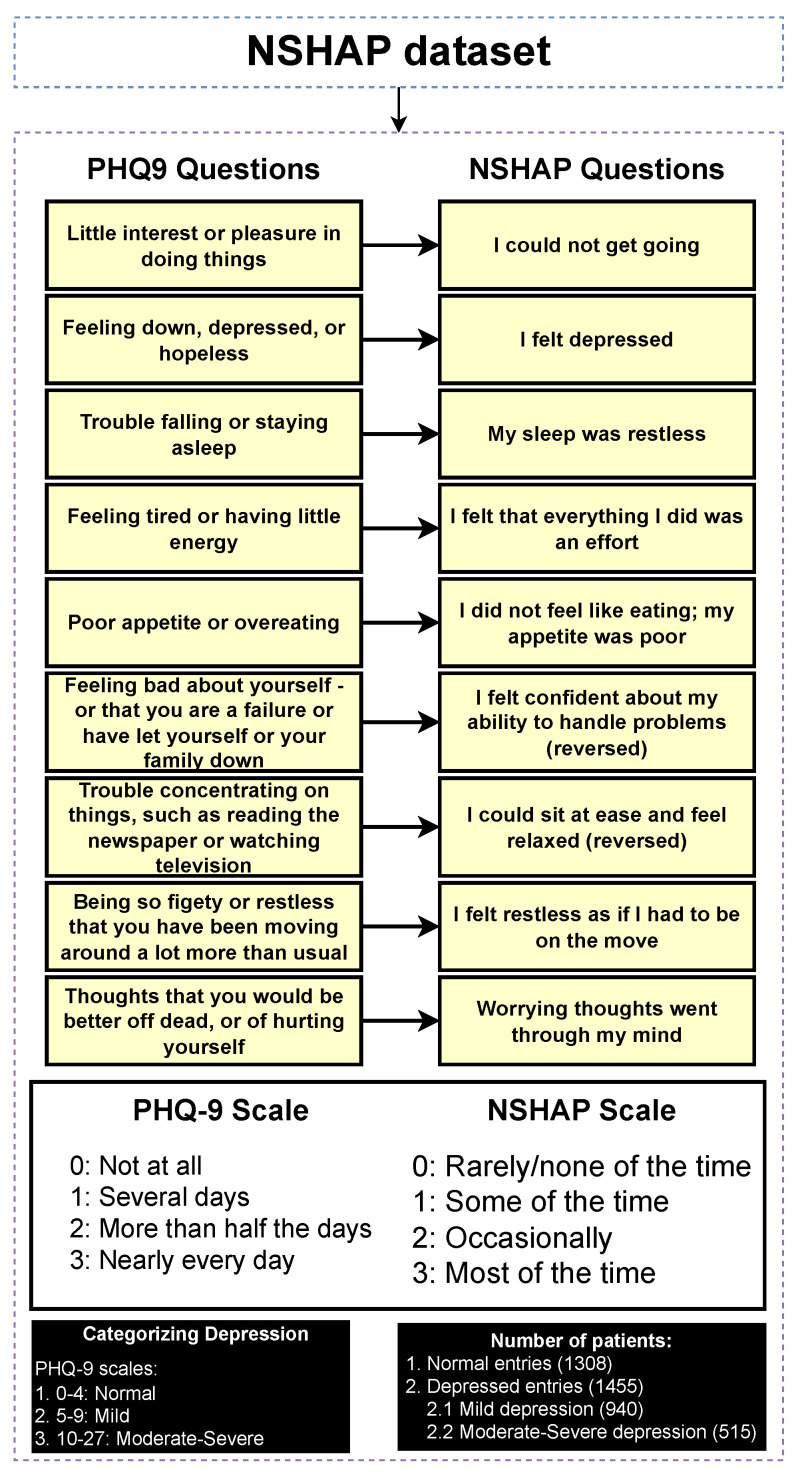
Categorizing entries into depression categories (normal, mild depression, moderate–severe depression).

The NSHAP dataset focuses on the general well-being of older Americans. Given its broad scope, the dataset does not include a specific depression categorization, as it covers a wide range of topics. However, it contains mental health questions that assess the mental health of participants in various categories. To address this, we can take advantage of PHQ-9, a widely recognized tool used to detect, diagnose, and measure the severity of depression. The PHQ-9 consists of nine questions that evaluate the frequency of depressive symptoms over the past two weeks, with responses ranging from “0” (not at all) to “3” (nearly every day). The total score ranges from 0 to 27, with higher scores indicating a higher severity of depression [32]. Figure 3 illustrates the mapping process in which each PHQ-9 question is matched to the corresponding questions of the NSHAP dataset that are as similar as possible. The response options (0–3) are aligned with the PHQ-9 scale, allowing for a coherent categorization of entries based on the PHQ-9 framework. The categorization of the entries was performed using the following mapping: normal (0–4), mild depression (5–9), and moderate to severe depression (10–27), based on the original categorizations of PHQ-9. Subsequently, the detection layer is designed to distinguish between normal and depressed individuals, where the depressed category includes mild and moderate–severe depression. The severity prediction layer, which focuses solely on depressed individuals, differentiates between mild and moderate–severe cases. The regression layer uses the entire dataset to predict the PHQ-9 scores of individuals.

Table 1 presents the results of a chi-square test comparing a selection of categorical features between normal and depressed individuals. The results show that several features have *p*-values ≤ 0.001, indicating a significant association between these features and the depression categories, thereby supporting the effectiveness of the categorization process. This is consistent with intuitive expectations, as features such as “self-rated general happiness” display very low *p*-values, suggesting a strong correlation with depression status. Thus, the categorization approach appeared to be valid and aligned with the data. Additionally, as part of further exploratory analysis, a comparison of numerical features between depressed and normal individuals is provided in Supplementary Table S15.

### 3.4. Data Pre-Processing

After certain entries and features are excluded, the data must undergo pre-processing to ensure that they are suitable for model processing. This preprocessing includes encoding categorical labels and imputing missing values to create a complete and analyzable dataset.

#### 3.4.1. Label Encode

There are numerical and categorical features in our tabular dataset. Since many models cannot process non-integer values, label encoding is necessary for the categorical features. Label encoding was chosen due to its simplicity and efficiency, particularly given that many of the categorical features are ordinal, such as scales for mental health questions. Additionally, for non-ordinal features, most are binary, making label encoding a suitable and straightforward choice.

#### 3.4.2. Data Imputation

Certain entries in the dataset exhibit missing values in various features. To address these gaps, median imputation within their specific categories has been identified as the most effective method based on empirical trials. This technique ensures that the imputed values accurately reflect their respective categories, thereby enhancing the overall integrity of the dataset. Imputing missing values is important as these features contribute to the model’s robustness and generalizability. Without imputation, the model could be forced to discard incomplete data, potentially leading to reduced sample size and introducing bias. This could negatively impact the model’s performance, especially in capturing key patterns and relationships within the data.

#### 3.4.3. Data Preparation

This section covers data splitting, data balancing, and data normalization. These are essential steps in developing a robust and accurate ML model.

#### 3.4.4. Data Splitting

The dataset is divided into 70% for training and 30% for testing. This separation is conducted prior to balancing and normalization to prevent data leakage. The division process is repeated 10 times, and the average results, along with standard deviations, are reported to ensure the generalization of the model.

#### 3.4.5. Data Balancing

The dataset is relatively balanced, with the detection layer showing a ratio of approximately 1.11:1 (depressed to normal) and the severity prediction layer showing a ratio of approximately 1.83:1 (mild to moderate–severe). However, data balancing was still performed to ensure the model is both robust and fair, preventing bias towards the more prevalent class. To achieve this, the Synthetic Minority Over-sampling Technique (SMOTE), a method that generates synthetic samples for the minority class to balance the dataset, was applied to the training data alone [60]. Additionally, random undersampling was performed on the test data to ensure that accuracy metrics reflect a fair distribution of categories, allowing for an accurate assessment of the model’s performance across both categories.

#### 3.4.6. Data Normalization

After splitting and balancing the dataset, normalization is performed using the min–max scaling technique to transform the data into a range with a mean of zero and a standard deviation of one. The scaler is fitted on the training data and subsequently applied to the testing data without refitting to prevent data leakage issues.

### 3.5. Depression Detection and Severity Prediction Layers

In this stage, a comparative performance analysis was conducted across various ML models. Initially, classical ML models were employed, including Decision Tree (DT), Logistic Regression (LR), Naive Bayes (NB), K-Neighbors (KN), Multilayer Perceptron (MLP), and Support Vector Classification (SVC). Following this, static ensemble models such as Random Forest (RF), XGBoost (XGB), Gradient Boosting (GB), AdaBoost (AB), CatBoost (CB), LightGBM (LGBM), and Voting Classifier (Vot) were evaluated. Finally, state-of-the-art DES algorithms were evaluated, including KNORAE, KNORAU, KNOP, DESMI, METADES, DESKNN, and DESP, along with their FIRE-enhanced versions: FIRE-KNORA-U, FIRE-KNORA-E, FIRE-METADES, FIRE-DESKNN, FIRE-DESP, and FIRE-KNOP. The FIRE framework enhances DES by focusing on classifiers capable of accurately distinguishing between ambiguous samples of different classes (frienemies) in indecision regions, thus improving overall classification performance [56].

While many classical ML models and static ensemble techniques have been applied to the field of depression detection and severity prediction [16,18,61], there has been limited exploration of DES. Static ensemble models generally outperform individual base classifiers [28], which is why they are often preferred. However, DES differs by selecting the most competent base classifiers for each new test sample in real-time, potentially improving accuracy [28]. Despite the potential of dynamic ensemble models [26,62,63], they remain underexplored in depression detection, particularly within the NSHAP dataset, where they have not, to our knowledge, been applied. Therefore, this study proposes the use of DES to improve the performance of the AI model in this field. We comprehensively evaluate classical, static, and dynamic models using default hyperparameters, optimized hyperparameters, with feature selection, and without feature selection, providing a detailed performance comparison.

### 3.6. PHQ-9 Scale Prediction Layer

The confirmation layer in the proposed framework serves as a regression layer aimed at predicting individual PHQ-9 scores. As its primary function is to provide additional validation and robustness to the predictions made by the main layers, only static ensemble regression algorithms are employed at this stage. The regression models utilized in this layer include CatBoost Regressor (CBR), XGBoost Regressor (XGBR), LightGBM Regressor (LGBMR), Gradient Boosting Regressor (GBR), Random Forest Regressor (RFR), Extra Trees Regressor (ETR), and AdaBoost Regressor (ABR).

### 3.7. Explainable Artificial Intelligence

XAI fosters transparency, trust, and ethical practices in AI systems. Transparent AI is essential for users and stakeholders to understand and trust decision-making processes, particularly in high-stakes domains such as healthcare. XAI addresses this need by providing interpretable insights into how AI models arrive at their predictions, enabling users to comprehend and trust the technology’s outcomes. This transparency is also crucial for ensuring ethical AI practices, such as fairness, accountability, and responsibility. It is challenging to uphold these ethical principles without clear insights into how AI models function. Moreover, XAI facilitates regulatory compliance, with frameworks such as the General Data Protection Regulation mandating explanations for decisions made by automated systems, ensuring the transparency necessary for legal adherence [64]. In the medical domain, XAI is particularly indispensable. According to Chaddad et al. (2023), adopting interpretable and transparent AI systems in healthcare is necessary to gain the trust of medical professionals and patients. XAI techniques such as feature visualization, saliency maps, and DTs have been successfully employed to enhance the interpretability of AI models in diagnostics, treatment planning, and patient management. This transparency is crucial to effectively integrate AI into clinical practice and ensure that clinicians can trust and rely on AI-assisted decision-making [65]. In mental health, XAI is gaining traction as a tool to enhance the precision and accessibility of mental health assessments and interventions. Byeon (2023) highlights how XAI, particularly through methods like SHapley Additive exPlanations (SHAP) and Local Interpretable Model-Agnostic Explanation (LIME), is being used to predict depression and assist in expert decision-making. By improving the interpretability of AI models in psychiatric applications, XAI plays a crucial role in increasing the acceptance of AI in mental health, particularly in identifying high-risk individuals and guiding treatment decisions [66].

Given the growing importance of XAI in promoting ethical AI development, ensuring regulatory compliance, and fostering trust, its incorporation into ML applications is essential. Explainability techniques provide deeper insights into how AI models generate predictions, building user confidence in the models’ outputs. Specifically, XAI allows for a clear understanding of the factors that drive the model’s predictions, particularly in identifying depression and assessing its severity in different stages of the framework. In the proposed framework for depression detection and severity prediction, various explainability techniques were applied to visualize and interpret the best-performing models at each layer. SHAP summary plots were generated to offer a global view of feature importance, detailing how specific features influence the model’s overall predictions. SHAP beeswarm plots complemented this by showing the distribution of feature impacts across individual instances. In addition, DT visualizations were employed to explain the hierarchical structure of decision-making, with decision-tree rule paths providing step-by-step insight into how individual predictions are made. For more instance-specific analysis, waterfall plots were created to break down feature contributions for both depression detection and severity prediction. These explainability techniques provide a comprehensive understanding of the key factors that influence the predictions of the model, providing critical information on the presence and severity of depression. By improving transparency and trust in the model, XAI facilitates the responsible deployment of AI systems in clinical and real-world settings, ensuring that these tools can be used confidently by healthcare professionals and end users alike.

## 4. Experimental Setup

The experiments in this study were performed on a system with the following specifications: an AMD Ryzen 9 5900HS CPU running at 3.3 GHz and 16 GB of RAM. The operating system used was Windows 11. The software environment included Python 3.12.3 with key libraries such as Imbalanced-learn 0.12.2, Numpy 1.26.4, Orange3 3.37.0, Pandas 2.2.2, Scikit-learn 1.4.2, and Matplotlib 3.8.4. The code of all experiments is available at https://github.com/InfoLab-SKKU/DES4Depression (accessed on 25 September 2024) and data are available at https://doi.org/10.3886/ICPSR20541.v10 (accessed on 25 April 2024).

### 4.1. Performance Evaluation Metrics

To assess the performance of the models for the detection and severity prediction layers, a comprehensive evaluation using various performance metrics was conducted, including accuracy, precision, recall, F1-score, and AUC. For regression models, metrics include RMSE, MAE (Mean Absolute Error), and $R^{2}$ (R-squared) to evaluate regression models.

### 4.2. Experimental Roadmap for the Detection and Severity Prediction Layers

In both the detection and severity prediction layers, three sublayers were implemented sequentially: classical ML, followed by static ensemble ML, and then dynamic ensemble models. These sublayers were applied in succession, with the best-performing models from each preceding sublayer used for feature selection in the following layer. Initially, classical ML models were employed, utilizing the top 200 features identified through correlation analysis. The most optimal classical ML model was then selected for feature selection for the static ensemble layer, narrowing down to the top 150 features. Subsequently, the best static ensemble model was used to identify the top 50 features of the dynamic ensemble layer. A list of all classical ML models, static ensemble models, and dynamic ensemble models can be found in Section 3.5. The sublayers of the classical models and static ensemble models underwent three testing conditions: without feature selection and without hyperparameter optimization, with feature selection but without hyperparameter optimization, and with both feature selection and hyperparameter optimization. The latter condition was used to determine the “best model” for feature selection in the subsequent layer. For the dynamic ensemble sublayer, only one testing condition was implemented, where all base classifiers were optimized, and feature selection was made. All base classifiers utilized within the dynamic ensemble sublayer correspond to the classifiers implemented in both the classical and static ensemble approaches. These base classifiers have undergone hyperparameter optimization to ensure optimal performance when incorporated into the dynamic ensemble sublayer. For the purpose of a fair comparison, the dynamic ensemble methods themselves are employed with default parameter settings. Hyperparameter optimization of the base classifiers was based on Bayesian optimization (Bayes search). It is important to note that hyperparameter optimization was performed subsequent to the feature selection step. The search spaces for optimizing hyperparameters of classical models can be found in Supplementary Listing S1, while the search spaces for optimizing hyperparameters of static ensemble models can be found in Supplementary Listing S2. Ultimately, the model demonstrating the highest performance was selected and further enhanced with XAI. Note that it is challenging to obtain feature importance with DES methods. Hence, the best classical ML or static ensemble model will be used to generate XAI.

### 4.3. Experimental Roadmap for the Scale Prediction Layer

A static ensemble approach will be employed utilizing several static ensemble regressors, a list of which is stated in Section 3.6. The study will be conducted in two phases: initially without feature selection and hyperparameter optimization, and subsequently with the implementation of both feature selection and hyperparameter optimization. The best model was then used in XAI. Feature selection was completed by the best model from the detection layer, while hyperparameter optimization was completed through Bayes search. The search spaces for optimizing hyperparameters of regressors can be found in Supplementary Listing S3.

### 4.4. Experimental Roadmap for XAI

Model explainability is conducted separately for all three layers. For the two primary layers—detection and severity prediction—both global explainability and local-instance explainability methods are employed. In the case of global explainability, the best-performing model from either the classical or static ensemble models is selected to generate SHAP summary plots and SHAP beeswarm plots. These visualizations provide insights into feature importance, illustrating how individual features contribute to the presence of depression in the detection layer or to the severity of depression in the severity prediction layer. Additionally, a DT visualization is created to demonstrate the decision-making process of the DT model. For local-instance explainability, waterfall plots are utilized to illustrate the model’s decision-making process for individual cases. In the detection layer, waterfall plots are generated to compare normal versus depressed individuals, while in the severity prediction layer, they compare mild versus moderate–severe depression cases. Furthermore, several sequential decision-making paths from a DT will be shown for both layers to see how a DT makes its decision when predicting the presence of depression or the severity of depression in an individual. In the confirmation layer (regression), only local-instance explainability is applied, specifically through the use of waterfall plots. These plots are generated for two extreme cases: a PHQ-9 score of 0 (indicating no depression) and a PHQ-9 score of 27 (indicating severe depression), providing detailed insights into the factors influencing the lowest and highest possible depression scores.

## 5. Results and Discussion

### 5.1. Results of Depression Detection

This section provides a comprehensive analysis of the outcomes derived from the detection layer experiments (i.e., normal individuals against depressed individuals). The discussion is structured into three subsections: classical ML models, static ensemble models, and dynamic ensemble models. The test results are collected and reported for all models. A 10-fold cross-validation is used for models’ optimization to ensure the robustness and stability of the results, and a holdout test set is employed to measure the generalization performance. The test results are presented as mean ± standard deviation to accurately convey the variability in the performance of the models. Accuracy and F1-score are utilized as primary metrics for the evaluation, providing a consistent baseline for comparison across different models. The findings of these evaluations not only highlight the effectiveness of each model but also inform the selection of features and optimization strategies for subsequent layers in the experimental roadmap.

#### 5.1.1. Classical ML Models

The performance of classical ML models is evaluated under three distinct conditions: without feature selection and hyperparameter optimization (refer to Supplementary Table S1), with feature selection only (refer to Supplementary Table S2), and with both feature selection and hyperparameter optimization (refer to Table 2). The feature selection process for this layer utilizes the top 200 features identified via the correlation operation. As anticipated, the performance of all classical ML models improved following the implementation of feature selection and hyperparameter optimization. The SVC model achieved the highest accuracy and F1-score (i.e., 81.47% ± 1.25% and 81.45% ± 1.24%, respectively).

**Figure 4:**
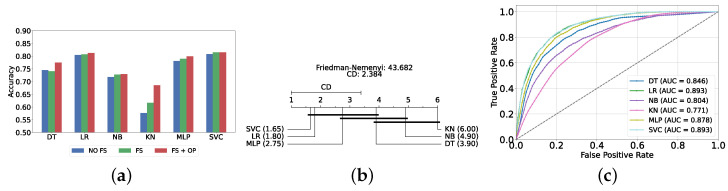
Performance comparison of different classical classifiers at the detection layer. Abbreviations: feature selection (fs); hyperparameter optimization (op); critical difference (cd); area under curve (auc). (**a**) Performance of classical classifiers with and without feature selection and optimization (detection layer). (**b**) Comparison of classical classifiers based on the Friedman test (detection layer). (**c**) AUC scores for classical classifiers with feature selection and hyperparameter optimization (detection layer).

Figure 4a provides a summary of this stage, illustrating the performance of classical ML models with and without the application of feature selection and hyperparameter optimization steps. As observed, the performance of all models, except for DT, improves after feature selection and further increases following hyperparameter optimization (including DT). This demonstrates the significance of these steps in optimizing model performance. Figure 4b shows the Friedman–Nemenyi test, revealing that SVC, LR, and MLP are statistically similar in performance, while these models are distinct from KNN, NB, and DT. Lastly, Figure 4c depicts the Receiver Operating Characteristic (ROC) curves along with their respective AUC scores, further evidencing the classifiers’ effectiveness in differentiating classes.

#### 5.1.2. Static Ensemble Models

In this section, we evaluate the performance of static ensemble models. Similar to the previous section, we assessed the performance of static ensemble ML models under three different conditions: without feature selection and hyperparameter optimization (refer to Supplementary Table S3), with feature selection only (refer to Supplementary Table S4), and with both feature selection and hyperparameter optimization (refer to Table 3). For feature selection, the top 150 features were identified using LR, as it is the second-best model evaluated in the previous classic ML models experiment. This choice is due to the difficulty in obtaining feature importance scores for SVC, which does not inherently provide a straightforward method for feature importance extraction.

**Figure 5:**
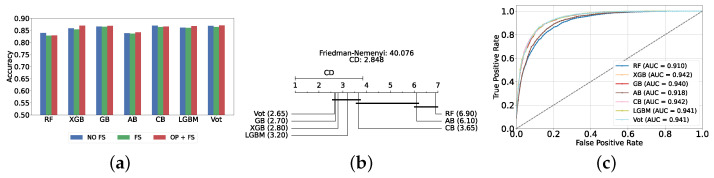
Performance comparison of different static ensemble classifiers at the detection layer. (**a**) Performance of static ensemble classifiers with and without feature selection and optimization (detection layer). (**b**) Comparison of static ensemble classifiers based on the Friedman test (detection layer). (**c**) AUC scores for static ensemble classifiers with feature selection and hyperparameter optimization (detection layer).

The most effective static ensemble model identified was the Vot classifier, which achieved an accuracy and F1-score of 87.08% ± 1.06% and 87.08% ± 1.06%, respectively. The Vot classifier excels because it leverages the strengths of multiple base models, combining their predictions to produce a more robust and accurate final decision. Moreover, this represents a significant improvement over the SVC model. Figure 5a provides a comparative analysis of model performance with and without the application of feature selection and hyperparameter optimization. It should be noted that some models experienced a decrease in accuracy following feature selection (without hyperparameter optimization), suggesting that a larger feature set may sometimes be beneficial. However, after optimizing hyperparameters, all models showed an increase in accuracy, indicating that optimizing hyperparameters for the new feature set is crucial for improving performance. Furthermore, all static ensemble models outperformed all classical ML models, highlighting the superiority of static ensemble methods over classical approaches. Figure 5b shows that Vot, GB, XGB, LGBM, and CB exhibit similar statistical performance, distinct from AB and RF, with RF being the lowest-ranked model. Finally, Figure 5c depicts the ROC curves alongside their corresponding AUC scores.

#### Comparison of Classic and Static Ensemble Classifiers

**Figure 6:**
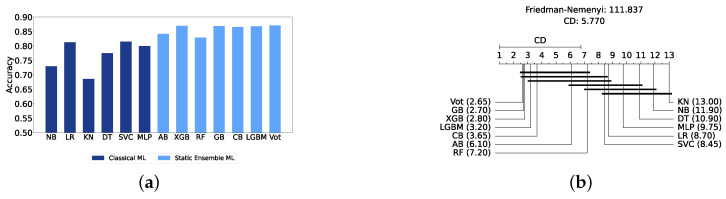
Performance comparison between different classic and static classifiers at the detection layer. (**a**) Performance metric comparison between classic and static ensemble classifiers (detection layer). (**b**) Comparison of classic and static ensemble classifiers based on the Friedman test (detection layer).

We present a comparison of classical and static ensemble models based on their accuracy, as illustrated in Figure 6a. It is noteworthy that all static ensemble models outperformed the classical ML models. The KNN model demonstrated the poorest performance among all models. Conversely, several static ensemble models, including Vot, LGBM, CB, GB, and XGB, exhibited similar high performance, with Vot being the highest-performing one. These results are further evidenced by the Friedman–Nemenyi test results illustrated in Figure 6b, which highlight Vot as the top performer with an average rank of 2.65, while KNN ranks the lowest with an average rank of 13.00.

#### 5.1.3. Dynamic Ensemble ML Models

In this section, we explore the performance of dynamic ensemble models toward the detection layer. It is worth mentioning that, in this evaluation, only 50 features were utilized, selected using XGB due to its superior performance in feature selection, except for the Vot classifier, for which feature importance could not be determined easily.

The construction of dynamic ensembles requires the selection of the optimal number and types of base classifiers. In particular, the list of base classifiers remains consistent with those optimized in previous sections. In this section, the performance of these ensembles was evaluated using the top three, four, five, and all six base classifiers, evaluated using twelve DES techniques.

#### Results of DES with a Pool of Classical Classifiers

**Figure 7:**
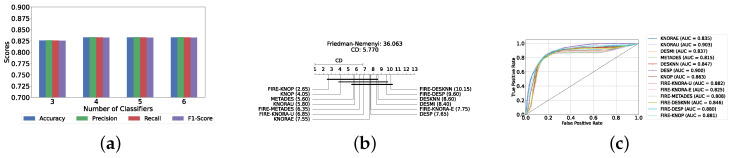
Performance comparison of different DES classifiers with classical classifiers at the detection layer. (**a**) Comparison of FIRE-KNOP with classical classifiers pool with different numbers of base classifiers (detection layer). (**b**) Comparison of DES classifiers with a pool of 6 classical classifiers based on the Friedman test (detection layer). (**c**) AUC scores for DES classifiers with a pool of 6 classical classifiers (detection layer).

With a pool of optimized classical ML classifiers, the use of the six base classifiers results in the highest precision. The best performance was achieved with the FIRE-KNOP method, which achieved an accuracy and F1-Score of 83.28% ± 1.60% and 83.27% ± 1.60% respectively. Detailed metrics for all DES techniques employing six classical classifiers are presented in Table 4. The enhanced performance of FIRE-KNOP can be attributed to the incorporation of the FIRE capability, which implements dynamic frienemy pruning within the KNOP algorithm [56]. Figure 7a illustrates the performance of the FIRE-KNOP model with varying numbers of base classifiers. The results indicate that the performance metrics remain consistent for configurations with 4, 5, and 6 base classifiers, while a slight decline in performance is observed when the number of base classifiers is reduced to 3. Figure 7b presents the results of the Friedman–Nemenyi test, demonstrating that many classifiers are statistically similar in terms of performance. Notably, FIRE-KNOP emerges as the best-performing model, while FIRE-DESKNN is identified as the worst-performing model in the comparison. Lastly, Figure 7c displays the ROC curve along with the respective AUC scores. Details on the performance of DES models with different numbers of base classical classifiers can be seen in Supplementary Table S5.

#### Results of DES with a Pool of Static Ensemble Models

We explored the potential to improve the performance of DES algorithms by using static ensemble models as the classifier pool for DES models. This approach was motivated by the superior performance of the static ensemble models observed in earlier experiments.

**Figure 8:**
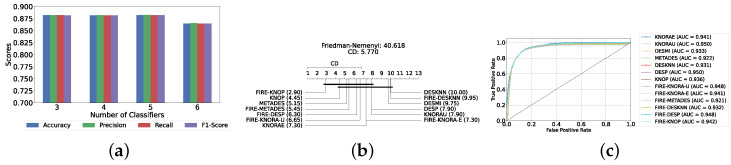
Performance comparison of DES classifiers with a static ensemble classifiers pool at the detection layer. (**a**) Comparison of FIRE-KNOP with a static ensemble classifiers pool with a different number of base classifiers (detection layer). (**b**) Comparison of DES classifiers with a pool of 5 static ensemble classifiers based on the Friedman test (detection layer). (**c**) AUC scores for DES classifiers with a pool of 5 static ensemble classifiers (detection layer).

Our evaluation indicates that the optimal configuration consists of using five base classifiers: XGB, GB, AB, CB, and LGBM. The most effective configuration involved using the FIRE-KNOP method with the five aforementioned base classifiers. This method consistently demonstrated superior performance, mirroring the results observed with DES using a pool of classical classifiers. Notably, the accuracies and F1-scores achieved with this configuration were significantly higher amongst all DES methods, reflecting the improved performance of using static ensemble models over classical models. Specifically, the FIRE-KNOP method achieved an accuracy and F1-Score of 88.21% ± 1.05% and 88.21% ± 1.05%, respectively. Detailed metrics for all DES models using five static classifiers can be found in Table 5. Figure 8a illustrates the performance of FIRE-KNOP with varying numbers of base classifiers. In this case, using three, four, or five classifiers resulted in similar performance metrics, while using six classifiers led to a decrease in performance. This contrasts with the results for DES using classical classifiers, suggesting that the number of classifiers may be less important than the selection of the classifiers themselves. Figure 8b presents the Friedman–Nemenyi test results, with FIRE-KNOP again emerging as the best-performing model and DESKNN as the worst, mirroring the trends observed with DES using classical classifiers. Finally, Figure 8c shows the ROC curve with the respective AUC scores. Details on the performance of DES models with a different number of base static classifiers can be seen in Supplementary Table S6.

#### Results of DES with a Mixed Pool of Classical and Static Ensemble Classifiers

**Figure 9:**
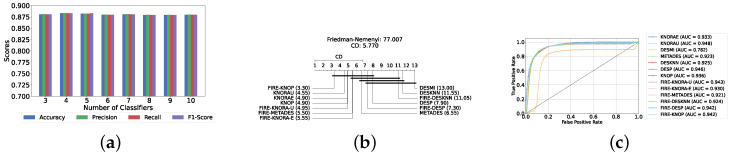
Performance comparison of DES classifiers with a mixed classifiers pool at the detection layer. (**a**) Comparison of FIRE-KNOP with a mixed classifiers pool with a different number of base classifiers (detection layer). (**b**) Comparison of DES classifiers with a pool of 4 mixed classifiers based on the Friedman test (detection layer). (**c**) AUC scores for DES classifiers with a pool of 4 mixed ensemble classifiers (detection layer).

A mixed pool of optimized classical and static ensemble classifiers was used to optimize the performance of DES models further. The aim was to enhance performance by leveraging the diversity offered by a combined pool. Various pool sizes were tested, ranging from four to ten base classifiers. The best performance across all experiments was achieved with a combination of three static ensemble classifiers and one classical classifier (XGB, CB, LGBM, SVC). Based on the optimal 3/1 combination of static and classical classifiers, we evaluated the performance of 12 different DES techniques. Consistently, the FIRE-KNOP method emerged as the best performer, achieving an accuracy and F1-Score of 88.33% ± 0.96% and 88.33% ± 0.96%, respectively. Additionally, the best-performing model exhibits the lowest standard deviation, indicating its greater stability and consistency in comparison to the other models. This result not only highlights the effectiveness of FIRE-KNOP but also demonstrates its stability, as evidenced by the lowest standard deviation compared to other DES methods for all metrics. Detailed metrics for all DES techniques using this configuration can be found in Table 6. Figure 9a illustrates the performance of FIRE-KNOP with varying numbers of base classifiers, demonstrating consistent metrics across configurations ranging from 3 to 10 base classifiers. This contrasts with the conclusions drawn from previous experimental stages, where the number of base classifiers did not significantly impact the performance of DES models, suggesting the potential insignificance of classifier quantity in this context. Figure 9b displays the Friedman–Nemenyi test, once again demonstrating that FIRE-KNOP is the best-performing model. However, DESMI is identified as the worst-performing model, differing from previous trends. Finally, Figure 9c displays the ROC curve with the respective AUC scores. Details on the performance of DES models with different numbers of base mixed classifiers can be seen in Supplementary Table S7.

### 5.2. Results of Depression Severity Prediction

In this section, we delve into the severity prediction layer, specifically focusing on differentiating between mild and moderate–severe depression. This task mirrors the preceding detection section in terms of methodology, involving the use of classical ML models, static ensemble models, and dynamic ensemble models. A 10-fold holdout testing technique was employed to ensure robust validation and accuracy, the F1-Score was chosen as the primary metric for evaluation, and Bayes search was utilized for hyperparameter tuning. All experimental procedures were conducted in alignment with those used in the detection layer, ensuring methodological consistency and reliability.

#### 5.2.1. Classical ML Models

**Figure 10:**
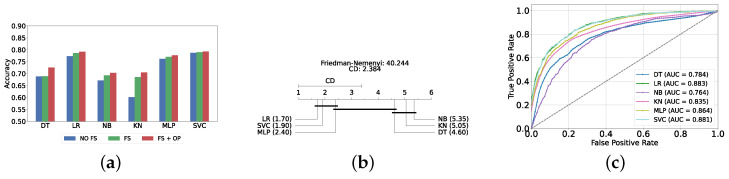
Performance comparison of different classical classifiers at the severity prediction layer. (**a**) Performance of classical classifiers with and without optimization (severity prediction layer). (**b**) Comparison of classical classifiers based on the Friedman test (severity prediction layer). (**c**) AUC scores for classical classifiers with feature selection and hyperparameter optimization (severity prediction layer).

The evaluation of the severity prediction layer follows the same process as the detection layer, using classical ML models under three conditions: without feature selection and hyperparameter optimization (refer to Supplementary Table S8), with feature selection only (refer to Supplementary Table S9), and with both feature selection and hyperparameter optimization (refer to Table 7). The feature selection process used the top 200 features identified by correlation, and the hyperparameters were subsequently optimized. The SVC remains the most effective model for the classical section in the severity prediction layer, achieving an accuracy and F1-Score of 79.26% ± 1.99% and 79.16% ± 2.04%, respectively. This performance is comparable to that of the detection task, although the accuracy is marginally lower by approximately 2%. This slight decrease in accuracy suggests that severity prediction might be more challenging than detection. Figure 10a provides an overview of this stage, illustrating the performance of ML models with and without the application of feature selection and hyperparameter optimization. As demonstrated, all models experienced a notable improvement in performance following feature selection, with an additional increase observed after hyperparameter optimization. The Friedman–Nemenyi test is shown in Figure 10b, indicating that LR, SVC, and MLP exhibit similar performance, which is statistically distinct from that of DT, KNN, and NB. This observation mirrors the results of the detection layer, potentially suggesting a consistent trend across these models. The ROC curves, along with the models’ respective AUC scores, are shown in Figure 10c.

#### 5.2.2. Static Ensemble ML Models

In this section, the performance of static ensemble models was evaluated for the depression severity prediction task, analogous to the depression detection discussed above. We evaluated the performance of static ML models under three conditions: without feature selection and hyperparameter optimization (refer to Supplementary Table S10), with feature selection only (refer to Supplementary Table S11), and with both feature selection and hyperparameter optimization (refer to Table 8). Similarly to the depression detection layer, the top 150 features were identified using LR.

Vot, once again, demonstrates superior performance, achieving the highest accuracy and F1-Score of 82.94% ± 1.78% and 82.76% ± 1.84%, respectively. A notable difference in this analysis is that AB emerged as the second highest performing model instead of XGB. In such case, AB will be utilized as the feature selector model for the DES layers, as it is difficult to obtain feature importance with the Vot model.

**Figure 11:**
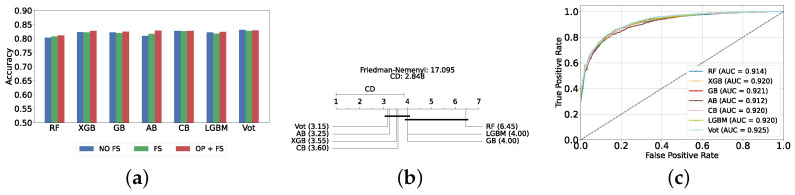
Performance comparison of different static ensemble classifiers at the severity prediction layer. (**a**) Performance of static ensemble classifiers with and without feature selection and optimization (severity prediction layer). (**b**) Comparison of static ensemble classifiers based on the Friedman test (severity prediction layer). (**c**) AUC scores for static ensemble classifiers with feature selection and hyperparameter optimization (severity prediction layer).

Figure 11a provides a comparative analysis of model performance with and without the application of feature selection and hyperparameter optimization. It is noteworthy that some models experienced a decrease in accuracy following feature selection (without hyperparameter optimization), suggesting that a larger feature set may sometimes be beneficial. However, after hyperparameter optimization, all models exhibited an increase in accuracy, indicating that optimizing hyperparameters for the new feature set is crucial for enhancing performance. Furthermore, all static ensemble models outperformed the classical ML models, as was similarly observed in the detection layer. This further reinforces the superiority of static ensemble models over classical ML approaches, highlighting their enhanced performance and effectiveness. Figure 11b indicates similar performance across all models, with the exception of RF, which emerges as the lowest-ranked model. This result is consistent with observations from the detection layer, further emphasizing this model’s comparatively weaker performance. Lastly, Figure 11c illustrates the ROC curves and their respective AUC scores.

#### Comparison of Classic and Static Ensemble Classifiers

**Figure 12:**
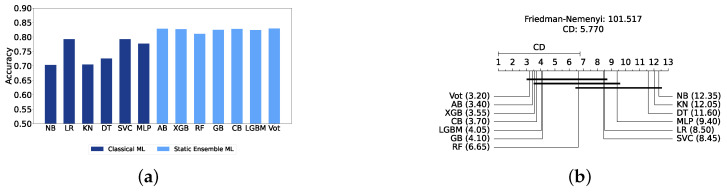
Performance comparison between different classic and static classifiers at the severity prediction layer. (**a**) Performance metric comparison between classic and static ensemble classifiers (severity prediction layer). (**b**) Comparison of classic and static ensemble classifiers based on the Friedman test (severity prediction layer).

This section presents a comparison of classical and static models based on their accuracy, as illustrated in Figure 12a, paralleling the analysis performed for the detection layer but with a lower average accuracy among models. This discrepancy signifies the increased difficulty in predicting the severity compared to detection. In particular, all static ensemble models outperformed the classical models. Specifically, the NB model demonstrated the poorest performance among the classical models in this context, while Vot still achieved the highest performance. These results are further supported by the Friedman–Nemenyi test results in Figure 12b, which highlights Vot as the top performer with an average rank of 3.35, while NB ranks the lowest with an average rank of 12.35.

#### 5.2.3. Dynamic Ensemble ML Models

In this section, we examine the performance of dynamic ensemble models in terms of the severity prediction layer. Only 50 features were utilized, selected using AB due to its superior performance in feature selection, except for the Vot classifier, for which feature importance could not be determined easily.

Similarly to the detection layer, the performance of these ensembles was evaluated using the top three, four, five, and all six base classifiers, evaluated using twelve DES techniques.

#### Results of DES with a Pool of Classical Classifiers

**Figure 13:**
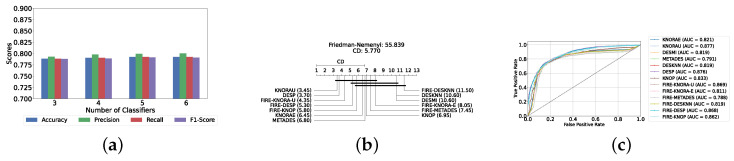
Performance comparison of different DES classifiers with classical classifiers at the severity prediction layer. (**a**) Comparison of KNORAU with classical classifiers pool with different numbers of base classifiers (severity prediction layer). (**b**) Comparison of DES classifiers with a pool of 5 classical classifiers based on the Friedman test (severity prediction layer). (**c**) AUC scores for DES classifiers with a pool of 5 classical classifiers (severity prediction layer).

Utilizing five base classifiers (DT, LR, KNN, MLP, and SVC) results in the highest performance. The best performance was achieved with the KNORAU method, achieving an accuracy and F1-Score of 79.26% ± 1.73% and 79.13% ± 1.77%, respectively. The DESP method yielded similarly strong results, though with a slightly higher standard deviation, indicating more variability in its performance. Detailed metrics for all DES techniques employing five classical classifiers are presented in Table 9. Figure 13a illustrates the performance of KNORAU with varying numbers of base classifiers, showing similar performances across all scenarios. Figure 13b displays the Friedman–Nemenyi test, indicating KNORAU as the best model and FIRE-DESKNN as the worst. Lastly, Figure 13c displays the ROC curve along with the respective AUC scores. Details on the performance of DES models with different numbers of base classical classifiers can be seen in Supplementary Table S12.

#### Results of DES with a Pool of Static Ensemble Models

Given that static ensemble classifiers have demonstrated superior performance compared to classical classifiers, analogous to their efficacy in the detection layer, we also explored the application of static ensemble methods within the DES layer. This approach mirrors the methodology employed in the detection layer, aiming to leverage the strengths of static ensembles for enhanced performance in the DES layer.

**Figure 14:**
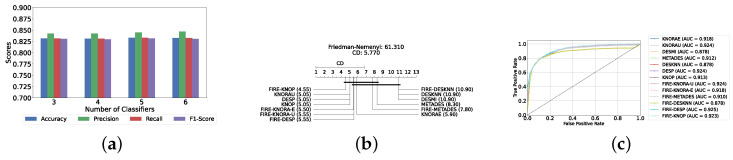
Performance comparison of DES classifiers with static ensemble classifiers pool at the severity prediction layer. (**a**) Comparison of FIRE-KNOP with a static ensemble classifiers pool with a different number of base classifiers (severity prediction layer). (**b**) Comparison of DES classifiers with a pool of 5 static ensemble classifiers based on the Friedman test (severity prediction layer). (**c**) AUC scores for DES classifiers with a pool of 5 static ensemble classifiers (severity prediction layer).

Similarly to the detection layer, our evaluation indicates that the optimal configuration consists of using five base classifiers: XGB, GB, AB, CB, and LGBM. The most effective configuration involved using the FIRE-KNOP method with the five aforementioned base classifiers. This parallels the findings in the depression detection layer, further highlighting the exceptional performance of the FIRE-KNOP method. Specifically, the FIRE-KNOP method achieved an accuracy and F1-Score of 83.32% ± 1.83% and 83.18% ± 1.86%, respectively. Detailed metrics for all DES models using five static classifiers can be found in Table 10. Figure 14a displays the performance of FIRE-KNOP with varying numbers of base classifiers, revealing similar performance across all configurations. When considered alongside the results from the previous DES with classical ML pool stage, this suggests that the number of base classifiers selected may have no statistically significant impact on the performance of the models, unlike in the detection layer’s DES with classical pool and DES with static pool stages. Figure 14b displays the Friedman–Nemenyi test, indicating FIRE-KNOP as the best model and FIRE-DESKNN as the worst. Lastly, Figure 14c shows the ROC curve with the respective AUC scores. Details on the performance of DES models with different numbers of base static classifiers can be seen in Supplementary File Table S13.

#### Results of DES with a Mixed Pool of Classical and Static Ensemble Models

**Figure 15:**
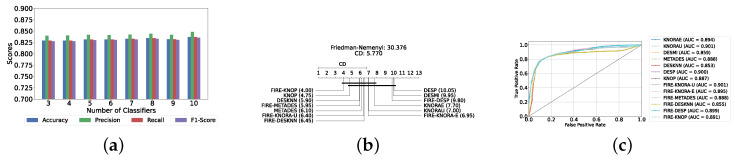
Performance comparison of DES classifiers with a mixed classifiers pool at the severity prediction layer. (**a**) Comparison of FIRE-KNOP with a mixed classifiers pool with a different number of base classifiers (severity prediction layer). (**b**) Comparison of DES classifiers with a pool of ten mixed classifiers based on the Friedman test (severity prediction layer). (**c**) AUC scores for DES classifiers with a pool of ten mixed ensemble classifiers (severity prediction layer).

Analogous to the detection layer, a mixed pool of optimized classical and static ensemble classifiers was used to improve performance by leveraging the diversity offered by a combined pool. Four to ten base classifiers were tested. The best performance across all experiments was achieved with a combination of six static ensemble classifiers and four classical classifiers (excluding NB and KNN). Consistently, the FIRE-KNOP method emerged as the best performer, achieving an accuracy and F1-Score of 83.68% ± 1.49% and 83.54% ± 1.53%, respectively. This result not only highlights the effectiveness of FIRE-KNOP but also demonstrates its stability, as evidenced by the lowest standard deviation compared to other accuracies and all other results. Detailed metrics for all DES techniques that use this configuration can be found in Table 11. Figure 15a illustrates the performance of FIRE-KNOP with varying numbers of base classifiers, showing statistically similar results across all configurations. This further reinforces the notion that the number of base classifiers has no significant impact on model performance. Figure 15b displays the Friedman–Nemenyi test, indicating FIRE-KNOP as the best model and DESP as the one with the worst performance. Finally, Figure 15c displays the ROC curve with the models’ respective AUC scores. Details on the performance of DES models with different numbers of base mixed classifiers can be seen in Supplementary Table S14. These findings underscore the superior performance of the FIRE-KNOP method, which consistently outperforms other DES techniques. The success of a mixed pool of classifiers suggests that incorporating a diverse set of classifiers leads to enhanced model performance. This pattern of results mirrors the observations in the detection layer, further affirming that mixed DES approaches tend to yield better outcomes, with FIRE-KNOP as the best method.

#### Summary of Best-Performing Models

Table 12 presents the best-performing models from all experiments conducted in both the detection and severity prediction layers. In the detection section, the FIRE-KNOP model with a mixed classifiers pool achieved the highest accuracy of 88.33% ± 0.96% and an F1-Score of 88.33% ± 0.96%. In the severity prediction section, the FIRE-KNOP model with a mixed classifiers pool produced the best metrics, with an accuracy of 83.68% ± 1.49% and an F1-Score of 83.54% ± 1.53%. Hence, it can be concluded that the FIRE-KNOP model, employing DES with a mix of classical and static classifiers, is the best-performing approach across both layers, surpassing all other techniques. Furthermore, these findings suggest that while the detection of depression is more straightforward, predicting the severity—distinguishing between mild and moderate to severe cases—presents a greater challenge, which is consistent with the literature on the difficulty of severity classification in depression, particularly when using scales like the PHQ-9 [67].

### 5.3. Results of PHQ-9 Depression Scale Prediction

Our experiments used the PHQ-9 scale to categorize both the presence and the severity of depression. Individuals diagnosed with depression using this method are typically assessed on a scale ranging from 0 to 27, with different score ranges corresponding to varying levels of severity. Given that a substantial number of patients have already been classified using this scale (in addition to the NSHAP dataset utilized in our study), regression analysis may be a viable approach to predict precise scores. This method seeks to estimate the exact scale score, where higher values indicate a greater severity of depression. More importantly, this approach can complement the two primary layers: detection and severity prediction, simultaneously determining the presence of depression and predicting its severity. This section explores the results of the regression analysis, followed by a detailed discussion of the findings.

#### 5.3.1. Static Ensemble Regression Models

In this study, we conducted two sets of experiments: one without feature selection and hyperparameter optimization, and another that incorporates both feature selection and hyperparameter optimization, as outlined in the experimental setup. For the latter set, feature selection will be performed with XGB, and hyperparameter optimization will be performed with a Bayesian search. These experiments were performed using seven static ensemble regression models.

#### Results Without Feature Selection and Hyperparameter Optimization

The average performance of the seven static ensemble models is presented in Table 13. The results indicate that the CBR model achieved superior performance, with an RMSE of 2.3549 ± 0.0500, an MAE of 1.8162 ± 0.0436, and an $R^{2}$ value of 0.7374 ± 0.0115.

#### Results with Feature Selection and Hyperparameter Optimization

**Figure 16:**
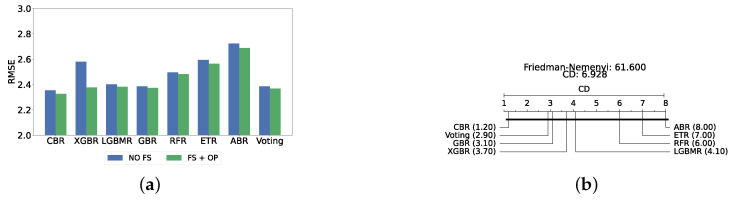
Performance comparison of static regressors at the scale prediction layer. (**a**) Performance comparison based on static regressors with and without feature selection and optimization (scale prediction layer). (**b**) Comparison of static regressors based on the Friedman test (scale prediction layer).

Upon optimizing the models using hyperparameter tuning and feature selection, we observed overall performance improvements across all models, and the CBR once again outperformed the others. The superior performance of CBR is evident in its performance metrics, with an RMSE of 2.3256 ± 0.0515, an MAE of 1.8099 ± 0.0494, and an $R^{2}$ value of 0.7439 ± 0.0115. This can be attributed to the unique approach of CBR in combining rule-based models with instance-based learning, which effectively captures complex patterns in the data [68]. Additionally, CBR’s ability to provide detailed and interpretable predictions makes it a robust choice for our regression tasks. Table 14 presents the performance metrics of all models post-optimization. Figure 16a provides a graphical comparison of the performance differences in static ensemble regressors using the RMSE metric, comparing results without feature selection to those with feature selection and hyperparameter optimization. As shown in the figure, all models improved after applying feature selection and hyperparameter optimization, highlighting the significance of these steps not only in classification layers but also in regression layers to optimize model performance. Figure 16b illustrates the results of the Friedman–Nemenyi test, revealing that CBR is the best-performing model, while ABR was the worst-performing model.

## 6. Model Explainability

Our experiments have yielded models capable of detecting depression presence, predicting its severity, and predicting the scale outcomes within the NSHAP dataset. However, understanding how these models make decisions and identifying the relevant features is crucial. This is where XAI comes into play, providing in-depth insights into the model’s operations and the factors influencing depression. XAI techniques improve transparency by elucidating the decision-making processes of the models. They allow us to identify which features are most important. By revealing these underlying mechanisms, XAI not only builds trust in the models but also offers valuable information on the factors that contribute to depression, ultimately informing better intervention strategies. Note that the detection layer’s XAI relies on the XGB static ensemble model to generate feature importance figures, while the severity prediction XAI utilizes the AB model. These models were chosen as they demonstrated the best performance during their respective experimental stages and facilitated the extraction of feature importance with ease. This approach was adopted because obtaining feature importance from dynamic ensemble models is more complex and less feasible.

### 6.1. Detection Layer XAI

**Figure 17:**
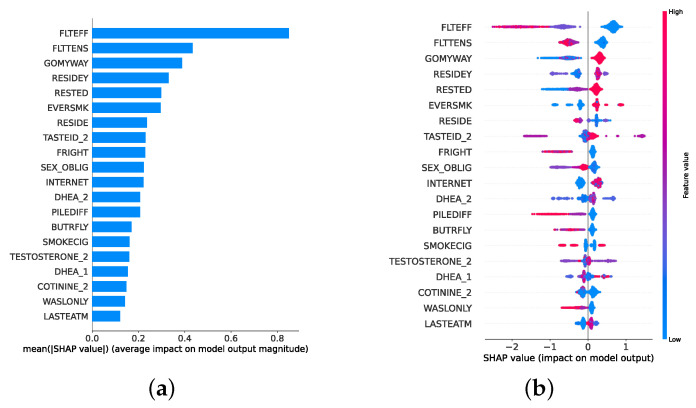
SHAP plots for feature importance in the detection model. (**a**) SHAP summary plot on feature importance for detection. (**b**) SHAP beesplot on feature importance for detection.

**Figure 18:**
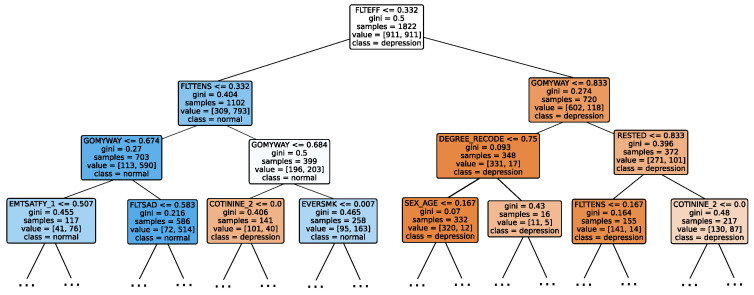
Decision tree classifier for detection.

Figure 17a presents a summary plot of the most significant features, while Figure 17b shows a beesplot illustrating the impact of each feature on predicting whether an individual is depressed or normal. Notably, many mental health questions, such as FLTEFF (“felt effortful when doing things“), FLTENS (“felt tense or wound up”), and GOMYWAY (“things are going my way”), are significant contributors. This aligns with the established practice of assessing depression through questionnaires such as PHQ-9 or BDI, which are reliable questionnaires for detecting the presence of depression in an individual [32,69]. For example, FLTEFF is a critical indicator. A lower value in FLTEFF (indicating less effort) correlates with being normal, whereas a higher value (indicating more effort) suggests depression. Among the features that are not related to mental health, an intriguing example is TASTEID_2 (identification when tasting). Studies have found that people with depression exhibit reduced olfactory performance, which in turn diminishes their ability to taste, as olfaction plays an important role in tasting [70,71]. Another interesting health examination feature is COTININE_2 (cotinine levels), which has been linked to depression, as higher cotinine levels may reduce depressive-like behaviors [72]. The DT analysis is shown in Figure 18, which further corroborates these findings, highlighting FLTEFF and GOMYWAY as crucial features. This is consistent with the summary plots, reinforcing their significance. Supplementary File Figure S1 provides two of the specific decision rule paths, tracing the progression from top-level features to final predictions, whether the individual is classified as normal or depressed. These decision paths offer a clearer understanding of how individual features contribute to the overall classification of the model, further illuminating the interpretability and transparency of the model.

**Figure 19:**
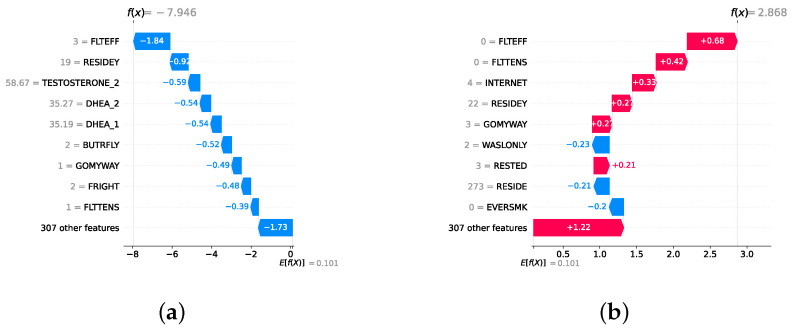
Waterfall plot for instances of depressed and normal individuals. (**a**) Waterfall plot for a predicted depressed individual. (**b**) Waterfall plot for a predicted normal individual.

Waterfall plots provide insights into local predictions, explaining how specific instances are classified as normal or depressed. In Figure 19, waterfall plots for a predicted depressed individual and a predicted normal individual can be seen. Figure 19a shows the depressed individual, in which high feelings of effortfulness to do things in life (FLTEFF) and low feelings of control (GOMYWAY) are key mental health indicators. Additionally, biological factors such as testosterone (especially in aging men [73] and DHEA levels [74] also influence the model’s prediction of depression. Conversely, in cases where the model predicts an individual to be normal, as shown in Figure 19b, the individual does not feel effortful when doing activities in life (FLTEFF) and does not feel much tension (FLTENS). These features consistently appear across various analyses, underscoring their importance in the model’s decision-making process.

### 6.2. Severity Prediction Layer XAI

**Figure 20:**
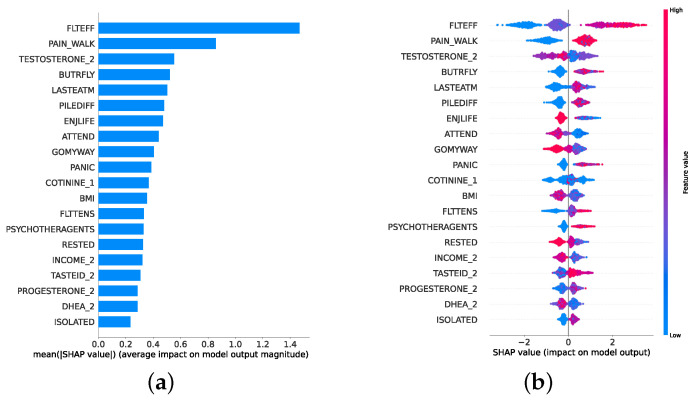
SHAP plots for feature importance in the severity prediction model. (**a**) SHAP summary plot on feature importance for severity prediction. (**b**) SHAP beesplot on feature importance for severity prediction.

**Figure 21:**
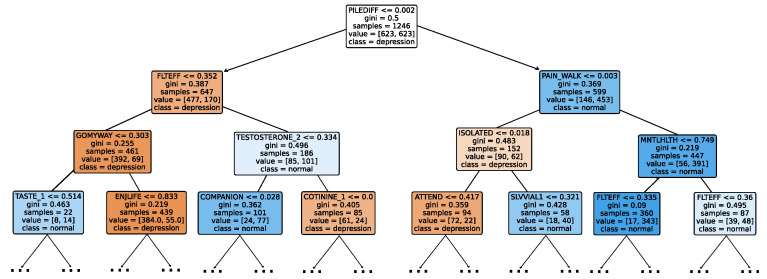
Decision tree classifier for severity prediction.

**Figure 22:**
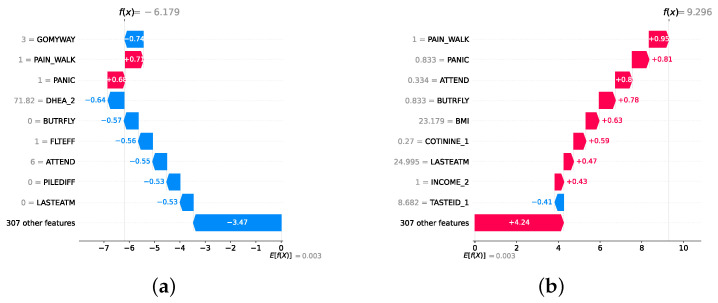
Waterfall plot for instances of mild and moderate–severe individuals. (**a**) Waterfall plot for a predicted mild individual. (**b**) Waterfall plot for a predicted moderate–severe individual.

The severity prediction layer is analyzed to determine the features for distinguishing mild and moderate–severe individuals. Figure 20a,b provide summary plots and beesplots, respectively. FLTEFF remains the most significant feature, with lower values indicating mild depression, and higher values indicating moderate to severe depression. However, additional features such as pain while walking (PWW), Body Mass Index (BMI), and income emerge as significant in the severity prediction layer, suggesting that the factors influencing the severity of depression differ from those determining the presence of depression. In examining these additional features, BMI, associated with being overweight or obese, has been found to increase the risk of depression, particularly in adults [75,76]. Additionally, research highlights a strong link between pain and depression, further suggesting medical evidence for our features such as PWW [77]. Similarly, the income-depression link is well established, with individuals of lower income being more likely to experience persistent depression [78]. However, the nature of these relationships—whether BMI, PWW, or income serve as markers or contributing factors—warrants further investigation. Figure 21, a DT, highlights PWW as the second most important feature. This suggests that higher levels of PWW correlate with increased severity of depression [79]. Supplementary File Figure S2 presents two specific decision rule paths, outlining the progression from top-level features to final predictions, whether the individual is classified as having mild or moderate–severe depression. Local instance-based analyses, shown in Figure 22a, further illustrate this. For a case of mild depression, key features include FLTEFF, although its impact is less pronounced, explaining the prediction as mild rather than moderate–severe. Conversely, in moderate–severe depression instances in Figure 22b, key features include PWW, PANIC (sudden feelings of panic), and ATTEND (attendance at meetings of organized groups in the past year [80]), which contribute to the classification as moderate–severe depression. While FLTEFF and several other features are common features across both severity prediction and detection layers, the detection layer reveals additional factors like PWW and BMI that are crucial for assessing depression severity. This differentiation underscores the complexity of depression and the need for tailored approaches in both diagnosis and treatment.

**Figure 23:**
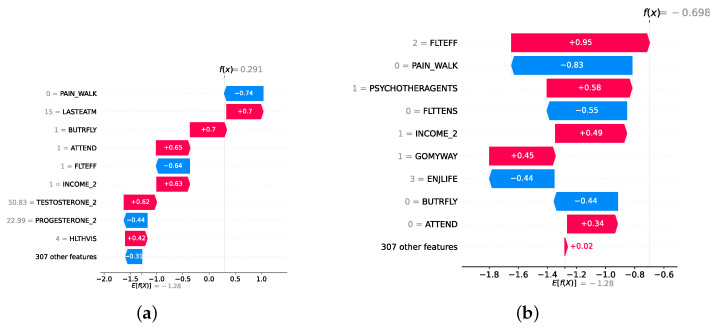
Waterfall plots for misclassified instances of mild and moderate–severe individuals. (**a**) Waterfall plot for a mild instance misclassified to moderate–severe. (**b**) Waterfall plot for a moderate–severe instance misclassified to mild.

As is apparent from the results section, predicting depression severity proves more challenging than detecting the presence of depression. To further understand this difficulty, we conducted an analysis of two local instances with misclassified predictions. Figure 23a shows an instance with a ground truth of mild severity, but the model misclassified it as moderate–severe. This misclassification can be attributed to the strong positive contributions of features like LASTEATM (recent eating habits) and TESTOSTERONE_2, which pushed the prediction towards higher severity despite the presence of mitigating features such as PWW and FLTEFF, which indicated lower severity. This suggests that the model may be overly sensitive to specific physiological or behavioral features that tend to drive predictions upward, leading to overestimation of severity in some cases. In contrast, Figure 23b depicts an instance where the ground truth was moderate–severe, but the model incorrectly predicted mild severity. Here, the feature FLTEFF contributed strongly towards higher severity, but the model’s attention to features such as PWW and FLTENS drove the prediction downwards. The model’s tendency to underweight certain psychological features and over-prioritize physical health factors appears to contribute to this underestimation. A potential improvement to address these misclassifications is the use of feature re-weighting or adjustment in feature importance during model training. By incorporating domain knowledge—such as emphasizing the significance of psychological or emotional health indicators in severity prediction—the model can be fine-tuned to avoid over-reliance on physical health metrics like pain-related features. Additionally, balancing the dataset or employing feature selection techniques to mitigate the dominance of certain features may also lead to better generalization in severity prediction. These adjustments may potentially help improve the model’s performance, particularly in accurately predicting depression severity.

### 6.3. Regression Layer XAI

**Figure 24:**
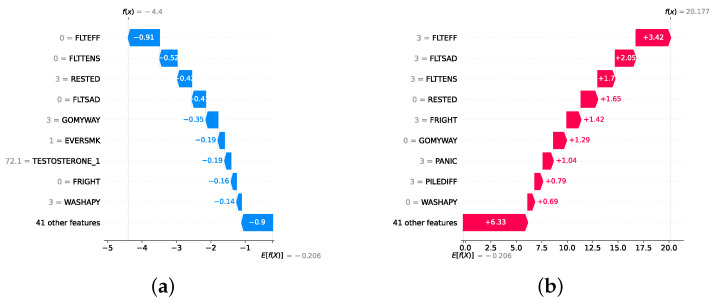
Waterfall plot of instances of lowest and highest scores. (**a**) Waterfall plot for score 0 (lowest). (**b**) Waterfall plot for score 27 (highest).

The regression layer analysis involves examining two waterfall plots: one with an extremely low depression score of 0 and another with an extremely high score of 27. This contrast allows for a detailed examination of both ends of the depression spectrum. In representing the 0 scores depicted in Figure 24a, we observe responses to mental health-related questions: a 0 FLTEFF, 0 FLTENS, and high scores in positive indicators such as RESTED (feeling rested). Notably, the feature EVERSMK (individuals with a history of cigarette smoking) leads the model to be more likely to classify such individuals as having a normal mental state rather than a depressed one. This may be attributed to the calming effects of nicotine and its potential use as an antidepressant [81], despite numerous studies in the literature associating smoking with depression [82].

In contrast, the waterfall plot depicting the score of 27 in Figure 24b shows elevated levels in all key mental health indicators: high FLTEFF, high FLTSAD (feelings of sadness) and low scores in indicators like RESTED (feeling rested) or WASHAPY (felt happy). This reinforces the importance of mental health questions in detecting depression, as these questions consistently emerge as significant predictors. This is why depression assessments often rely heavily on questionnaires such as the PHQ-9 and BDI that ask questions about mental health.

### 6.4. Top 50 Features in the Detection Layer and the Severity Prediction Layer

In this section, we identify the top 50 features by conducting 10 trials with the best static ensemble models (as it is difficult to obtain feature importances in DES methods) and selecting the most frequently occurring features. This approach provides clinicians with an abstract overview of key indicators for the presence and severity of depression. Supplementary File Figure S3 presents the 50 most frequent features identified for detection. These features encompass several categories, including mental health, relationship happiness, household income, and level of education. Supplementary File Figure S4 illustrates the top 50 features for predicting the severity of depression. In particular, there is a higher prevalence of health examination features in this analysis compared to the detection layer. Features such as blood pressure, BMI, and taste identification are more prominent, suggesting that severe depression may be associated with significant differences in these health metrics. In addition, social network support features, such as feelings of loneliness or social isolation, are more prevalent. Although there are many similarities between the two sets of features, the distinction highlights the complexity and multifaceted nature of depression.

In this study, the proposed model achieved the best results compared to the literature. The model provides accurate and personalized decisions. In addition, the model provided medically relevant XAI features that improved the trustworthiness of physicians. The resulting model is medically applicable in real medical settings and is expected to improve the detection capabilities of domain experts. Practical implications of the model in a real environment can be achieved by extending it in the following directions: (1) implementation of a clinical decision support system that smoothly integrates model decisions with XAI, (2) an external and clinical validation of the model to evaluate its generalization results based on domain experts usage and external datasets that evaluate the model’s real-world efficacy, (3) integration of the resulting systems with the hospital’s electronic health record ecosystem, and (4) extension of the current detection model to provide treatment plans based on severity prediction, which could optimize clinical resources and outcomes. The current study is based on structured data that provide a more applicable and cheaper method to detect depression. However, the fusion of multimodal data such as biomarkers, gyroscopic findings, and functional magnetic resonance images is expected to improve the performance and medical relevance of the resulting model. This integration of different data sources is expected to provide a deeper understanding of the disease, which results in new disease biomarkers that can be used by the domain expert in real medical settings.

## 7. Conclusions

This study presents a comprehensive framework to detect and predict the severity of depression using a multilayered approach that explores classical ML models, static ensemble models, and DES techniques. The primary objective is to improve the precision and interpretability of depression detection and severity assessment among older adults using the NSHAP dataset. Through rigorous experimentation, we demonstrated that dynamic ensemble models, particularly the FIRE-KNOP method, consistently outperformed classical and static ensemble models in both detection (normal vs. depressed) and severity prediction (mild vs. moderate–severe depression) tasks. The highest accuracy achieved in the detection layer was 88.33% ± 0.96% using the FIRE-KNOP method with a mixed pool of classical and static ensemble classifiers. In the severity prediction layer, the FIRE-KNOP method with a mixed set of classical and static ensemble classifiers also achieved an accuracy of 83.68% ± 1.49%. These results underscore the superior performance and robustness of dynamic ensemble techniques over traditional models. Furthermore, mixing classical and static ensemble classifiers in DES techniques proved to be the best approach because it leverages the strengths of both methods, enhancing overall model accuracy and robustness. The regression layer further enriched our analysis by predicting exact depression scores, which complements the other layers. The CBR model emerged as the most effective, achieving an RMSE of 2.3256 ± 0.0515. This highlights the potential of combining rule-based and instance-based learning methodologies to make precise predictions. After implementing all three layers, the detection layer enables us to predict whether an individual has depression with approximately 88% confidence. If depression is detected, the severity prediction layer can estimate the individual’s severity level with about 83% confidence. The regression layer then serves as a confirmation layer, predicting the exact PHQ-9 score with a margin of error of approximately 2.33 RMSE on a scale of 0 to 27. This allows for verification of whether the PHQ-9 score aligns with the predictions from the detection and severity prediction layers. The best models are selected to provide XAI features for decision-making. This process improves the understanding and transparency of the decision. We applied two well-known SHAP and DT techniques to provide global and local XAI features. The explainability consistently highlighted key features such as FLTEFF, TASTEID_2, COTININE_2, and GOMYWAY, which are medically critical to predicting depression. This study contributes to the field by providing a robust and interpretable framework for the assessment of depression by integrating dynamic ensemble models with XAI techniques.

## Figures and Tables

**Table 1:** Chi-square test on a selection of categorical features (normal and depressed).

Feature Description	Chi–Square Value	*p*–Value
Interviewer’s rating for interviewee’s posture	62.7900	$7.51\times 10^{-13}$
Happiness in current/past relationship	58.1990	$1.04\times 10^{-10}$
Numerical questions performance	18.9570	$1.34\times 10^{-5}$
Freq of internet usage	63.7290	$2.06\times 10^{-12}$
Self-rated general happiness	330.409	$2.97\times 10^{-70}$
Gender	26.6790	$2.40\times 10^{-7}$
Difficulty getting out of bed	126.823	$2.62\times 10^{-27}$
Disabled	81.7890	$1.51\times 10^{-19}$

**Table 2:** Classical classifier results with feature selection and hyperparameter optimization (detection layer).

Model	Accuracy	Precision	Recall	F1-Score
DT	0.7750±0.0162	0.7774±0.0154	0.7750±0.0162	0.7745±0.0166
LR	0.8126±0.0142	0.8140±0.0145	0.8126±0.0142	0.8124±0.0142
NB	0.7294±0.0187	0.7325±0.0190	0.7294±0.0187	0.7286±0.0187
KN	0.6860±0.0146	0.7363±0.0157	0.6860±0.0146	0.6683±0.0173
MLP	0.7995±0.0171	0.8002±0.0168	0.7995±0.0171	0.7993±0.0172
**SVC**	0.8147±0.0125	0.8158±0.0127	0.8147±0.0125	0.8145±0.0124

The bold row shows the best performing model. Abbreviations: Decision Tree (DT); Logistic Regression (LR); Naive Bayes (NB); K-Neighbors (KN); Multilayer Perceptron (MLP); Support Vector Classification (SVC).

**Table 3:** Static ensemble classifier results with feature selection and hyperparameter optimization (detection layer).

Model	Accuracy	Precision	Recall	F1-Score
RF	0.8288±0.0111	0.8290±0.0110	0.8288±0.0111	0.8287±0.0111
XGB	0.8698±0.0100	0.8700±0.0099	0.8698±0.0100	0.8697±0.0100
GB	0.8688±0.0130	0.8692±0.0130	0.8688±0.0130	0.8688±0.0130
AB	0.8419±0.0124	0.8424±0.0123	0.8419±0.0124	0.8419±0.0124
CB	0.8660±0.0099	0.8665±0.0098	0.8660±0.0099	0.8659±0.0099
LGBM	0.8679±0.0155	0.8683±0.0153	0.8679±0.0155	0.8678±0.0156
**Vot**	0.8708±0.0106	0.8712±0.0105	0.8708±0.0106	0.8708±0.0106

Abbreviations: Random Forest (RF); XGBoost (XGB); Gradient Boosting (GB); AdaBoost (AB); CatBoost (CB); LightGBM (LGBM); Voting Classifier (Vot).

**Table 4:** DES model results with all six base classical classifiers (detection layer).

Model	Accuracy	Precision	Recall	F1-Score
KNORAE	0.8202±0.0167	0.8207±0.0166	0.8202±0.0167	0.8201±0.0167
KNORAU	0.8285±0.0112	0.8301±0.0114	0.8285±0.0112	0.8283±0.0112
KNOP	0.8313±0.0186	0.8318±0.0184	0.8313±0.0186	0.8312±0.0186
DESMI	0.8237±0.0171	0.8254±0.0158	0.8237±0.0171	0.8234±0.0174
METADES	0.8290±0.0199	0.8295±0.0196	0.8290±0.0199	0.8290±0.0200
DESKNN	0.8207±0.0178	0.8231±0.0161	0.8207±0.0178	0.8203±0.0181
DESP	0.8259±0.0101	0.8267±0.0103	0.8259±0.0101	0.8258±0.0101
FIRE-KNORA-U	0.8243±0.0136	0.8249±0.0137	0.8243±0.0136	0.8242±0.0136
FIRE-KNORA-E	0.8191±0.0171	0.8195±0.0166	0.8191±0.0171	0.8190±0.0172
FIRE-METADES	0.8267±0.0199	0.8274±0.0193	0.8267±0.0199	0.8266±0.0201
FIRE-DESKNN	0.8176±0.0160	0.8207±0.0142	0.8176±0.0160	0.8171±0.0163
FIRE-DESP	0.8223±0.0107	0.8227±0.0105	0.8223±0.0107	0.8223±0.0108
**FIRE-KNOP**	0.8328±0.0160	0.8335±0.0160	0.8328±0.0160	0.8327±0.0160

**Table 5:** DES model results with five base static classifiers (detection layer).

Model	Accuracy	Precision	Recall	F1-Score
KNORAE	0.8790±0.0103	0.8794±0.0101	0.8790±0.0103	0.8790±0.0103
KNORAU	0.8785±0.0102	0.8789±0.0101	0.8785±0.0102	0.8785±0.0102
KNOP	0.8804±0.0110	0.8807±0.0109	0.8804±0.0110	0.8803±0.0110
DESMI	0.8751±0.0104	0.8760±0.0102	0.8751±0.0104	0.8751±0.0104
METADES	0.8810±0.0112	0.8814±0.0110	0.8810±0.0112	0.8810±0.0112
DESKNN	0.8747±0.0095	0.8756±0.0091	0.8747±0.0095	0.8747±0.0096
DESP	0.8785±0.0102	0.8789±0.0101	0.8785±0.0102	0.8785±0.0102
FIRE-KNORA-U	0.8789±0.0109	0.8793±0.0107	0.8789±0.0109	0.8789±0.0109
FIRE-KNORA-E	0.8790±0.0106	0.8794±0.0104	0.8790±0.0106	0.8790±0.0106
FIRE-METADES	0.8801±0.0110	0.8805±0.0108	0.8801±0.0110	0.8801±0.0110
FIRE-DESKNN	0.8750±0.0097	0.8759±0.0093	0.8750±0.0097	0.8749±0.0098
FIRE-DESP	0.8790±0.0110	0.8795±0.0108	0.8790±0.0110	0.8790±0.0110
**FIRE-KNOP**	0.8821±0.0105	0.8825±0.0104	0.8821±0.0105	0.8821±0.0105

**Table 6:** DES model results with a pool of four mixed classifiers (detection layer).

Model	Accuracy	Precision	Recall	F1-Score
KNORAE	0.8812±0.0101	0.8817±0.0100	0.8812±0.0101	0.8811±0.0101
KNORAU	0.8819±0.0101	0.8824±0.0100	0.8819±0.0101	0.8818±0.0101
KNOP	0.8817±0.0101	0.8821±0.0099	0.8817±0.0101	0.8817±0.0101
DESMI	0.8187±0.0110	0.8206±0.0111	0.8187±0.0110	0.8184±0.0110
METADES	0.8813±0.0118	0.8818±0.0116	0.8813±0.0118	0.8813±0.0118
DESKNN	0.8695±0.0133	0.8709±0.0126	0.8695±0.0133	0.8694±0.0133
DESP	0.8790±0.0106	0.8796±0.0104	0.8790±0.0106	0.8790±0.0107
FIRE-KNORA-U	0.8817±0.0109	0.8822±0.0107	0.8817±0.0109	0.8817±0.0109
FIRE-KNORA-E	0.8809±0.0102	0.8814±0.0101	0.8809±0.0102	0.8809±0.0102
FIRE-METADES	0.8817±0.0120	0.8822±0.0118	0.8817±0.0120	0.8817±0.0120
FIRE-DESKNN	0.8711±0.0123	0.8722±0.0118	0.8711±0.0123	0.8710±0.0124
FIRE-DESP	0.8793±0.0115	0.8798±0.0112	0.8793±0.0115	0.8793±0.0115
**FIRE-KNOP**	0.8833±0.0096	0.8838±0.0095	0.8833±0.0096	0.8833±0.0096

**Table 7:** Classical classifier results with feature selection and hyperparameter optimization (severity prediction layer).

Model	Accuracy	Precision	Recall	F1-Score
DT	0.7252±0.0235	0.7347±0.0228	0.7252±0.0235	0.7222±0.0245
LR	0.7919±0.0187	0.7955±0.0184	0.7919±0.0187	0.7913±0.0189
NB	0.7029±0.0283	0.7077±0.0282	0.7029±0.0283	0.7011±0.0289
KN	0.7048±0.0146	0.7689±0.0152	0.7048±0.0146	0.6860±0.0172
MLP	0.7768±0.0171	0.7844±0.0173	0.7768±0.0171	0.7753±0.0173
**SVC**	0.7926±0.0199	0.7980±0.0182	0.7926±0.0199	0.7916±0.0204

**Table 8:** Static ensemble classifier results with feature selection and hyperparameter optimization (severity prediction layer).

Model	Accuracy	Precision	Recall	F1-Score
RF	0.8110±0.0161	0.8332±0.0141	0.8110±0.0161	0.8077±0.0171
XGB	0.8271±0.0187	0.8393±0.0163	0.8271±0.0187	0.8255±0.0194
GB	0.8242±0.0203	0.8377±0.0167	0.8242±0.0203	0.8223±0.0212
AB	0.8284±0.0196	0.8367±0.0156	0.8284±0.0196	0.8272±0.0203
CB	0.8274±0.0211	0.8393±0.0181	0.8274±0.0211	0.8258±0.0219
LGBM	0.8235±0.0218	0.8386±0.0174	0.8235±0.0218	0.8214±0.0230
**Vot**	0.8294±0.0178	0.8431±0.0158	0.8294±0.0178	0.8276±0.0184

**Table 9:** DES model results with five base classical classifiers (severity prediction layer).

Model	Accuracy	Precision	Recall	F1-Score
KNORAE	0.7816±0.0187	0.7941±0.0187	0.7816±0.0187	0.7792±0.0193
**KNORAU**	0.7926±0.0173	0.7995±0.0165	0.7926±0.0173	0.7913±0.0177
KNOP	0.7819±0.0218	0.7907±0.0193	0.7819±0.0218	0.7802±0.0225
DESMI	0.7658±0.0210	0.7903±0.0171	0.7658±0.0210	0.7606±0.0225
METADES	0.7806±0.0211	0.7903±0.0193	0.7806±0.0211	0.7787±0.0217
DESKNN	0.7658±0.0210	0.7903±0.0171	0.7658±0.0210	0.7606±0.0225
DESP	0.7926±0.0181	0.7999±0.0167	0.7926±0.0181	0.7912±0.0187
FIRE-KNORA-U	0.7897±0.0181	0.7976±0.0183	0.7897±0.0181	0.7883±0.0183
FIRE-KNORA-E	0.7784±0.0190	0.7909±0.0183	0.7784±0.0190	0.7759±0.0197
FIRE-METADES	0.7790±0.0200	0.7887±0.0184	0.7790±0.0200	0.7771±0.0206
FIRE-DESKNN	0.7632±0.0206	0.7887±0.0155	0.7632±0.0206	0.7577±0.0222
FIRE-DESP	0.7874±0.0161	0.7961±0.0161	0.7874±0.0161	0.7858±0.0164
FIRE-KNOP	0.7858±0.0181	0.7943±0.0168	0.7858±0.0181	0.7842±0.0187

**Table 10:** DES model results with five base static classifiers (severity prediction layer).

Model	Accuracy	Precision	Recall	F1-Score
KNORAE	0.8319±0.0185	0.8445±0.0180	0.8319±0.0185	0.8304±0.0189
KNORAU	0.8329±0.0186	0.8446±0.0181	0.8329±0.0186	0.8314±0.0189
KNOP	0.8329±0.0184	0.8448±0.0178	0.8329±0.0184	0.8314±0.0187
DESMI	0.8177±0.0166	0.8391±0.0160	0.8177±0.0166	0.8148±0.0173
METADES	0.8277±0.0174	0.8431±0.0167	0.8277±0.0174	0.8258±0.0179
DESKNN	0.8177±0.0166	0.8391±0.0160	0.8177±0.0166	0.8148±0.0173
DESP	0.8329±0.0186	0.8446±0.0181	0.8329±0.0186	0.8314±0.0189
FIRE-KNORA-U	0.8326±0.0189	0.8443±0.0184	0.8326±0.0189	0.8311±0.0193
FIRE-KNORA-E	0.8319±0.0188	0.8447±0.0186	0.8319±0.0188	0.8303±0.0192
FIRE-METADES	0.8281±0.0172	0.8436±0.0164	0.8281±0.0172	0.8261±0.0177
FIRE-DESKNN	0.8177±0.0166	0.8391±0.0160	0.8177±0.0166	0.8148±0.0173
FIRE-DESP	0.8326±0.0189	0.8443±0.0184	0.8326±0.0189	0.8311±0.0193
**FIRE-KNOP**	0.8332±0.0183	0.8450±0.0177	0.8332±0.0183	0.8318±0.0186

**Table 11:** DES model results with a pool of ten mixed classifiers (severity prediction layer).

Model	Accuracy	Precision	Recall	F1-Score
KNORAE	0.8294±0.0171	0.8425±0.0164	0.8294±0.0171	0.8277±0.0175
KNORAU	0.8310±0.0194	0.8420±0.0187	0.8310±0.0194	0.8296±0.0198
KNOP	0.8348±0.0130	0.8469±0.0137	0.8348±0.0130	0.8334±0.0132
DESMI	0.8255±0.0184	0.8416±0.0171	0.8255±0.0184	0.8234±0.0191
METADES	0.8332±0.0138	0.8456±0.0139	0.8332±0.0138	0.8317±0.0142
DESKNN	0.8326±0.0176	0.8439±0.0168	0.8326±0.0176	0.8312±0.0180
DESP	0.8258±0.0174	0.8394±0.0163	0.8258±0.0174	0.8240±0.0180
FIRE-KNORA-U	0.8319±0.0191	0.8433±0.0179	0.8319±0.0191	0.8305±0.0196
FIRE-KNORA-E	0.8310±0.0163	0.8443±0.0159	0.8310±0.0163	0.8293±0.0167
FIRE-METADES	0.8335±0.0128	0.8461±0.0128	0.8335±0.0128	0.8320±0.0132
FIRE-DESKNN	0.8319±0.0172	0.8434±0.0166	0.8319±0.0172	0.8305±0.0176
FIRE-DESP	0.8261±0.0179	0.8395±0.0165	0.8261±0.0179	0.8244±0.0185
**FIRE-KNOP**	0.8368±0.0149	0.8479±0.0137	0.8368±0.0149	0.8354±0.0153

**Table 12:** Comparison of the best models from all experiments in the detection layer and the severity prediction layer.

Task	Experiments	Best Model	Accuracy	Precision	Recall	F1-Score
**Detection**	Classic	SVC	0.8147 ± 0.0125	0.8158 ± 0.0127	0.8147 ± 0.0125	0.8145 ± 0.0124
	Static	Vot	0.8708 ± 0.0106	0.8712 ± 0.0105	0.8708 ± 0.0106	0.8708 ± 0.0106
	DESw/Classic	FIRE-KNOP	0.8328 ± 0.0160	0.8335 ± 0.0160	0.8328 ± 0.0160	0.8327 ± 0.0160
	DESw/Static	FIRE-KNOP	0.8821 ± 0.0105	0.8825 ± 0.0104	0.8821 ± 0.0105	0.8821 ± 0.0105
	**DESw/Mix**	**FIRE-KNOP**	**0.8833 ± 0.0096**	**0.8838 ± 0.0095**	**0.8833 ± 0.0096**	**0.8833 ± 0.0096**
**Severity**	Classic	SVC	0.7926 ± 0.0199	0.7980 ± 0.0182	0.7926 ± 0.0199	0.7916 ± 0.0204
	Static	Vot	0.8294 ± 0.0178	0.8431 ± 0.0158	0.8294 ± 0.0178	0.8276 ± 0.0184
	DESw/Classic	KNORAU	0.7926 ± 0.0173	0.7995 ± 0.0165	0.7926 ± 0.0173	0.7913 ± 0.0177
	DESw/Static	FIRE-KNOP	0.8332 ± 0.0183	0.8450 ± 0.0177	0.8332 ± 0.0183	0.8318 ± 0.0186
	**DESw/Mix**	**FIRE-KNOP**	**0.8368 ± 0.0149**	**0.8479 ± 0.0137**	**0.8368 ± 0.0149**	**0.8354 ± 0.0153**

**Table 13:** Static ensemble regression model results without feature selection and hyperparameter optimization (scale prediction layer).

Model	RMSE	MAE	$R^{2}$
**CBR**	2.3549±0.0500	1.8162±0.0436	0.7374±0.0115
XGBR	2.5806±0.0628	1.9732±0.0483	0.6847±0.0147
LGBMR	2.4011±0.0499	1.8504±0.0387	0.7270±0.0128
GBR	2.3849±0.0619	1.8562±0.0553	0.7307±0.0127
RFR	2.4957±0.0690	1.9341±0.0615	0.7052±0.0116
ETR	2.5940±0.0622	2.0205±0.0603	0.6814±0.0128
ABR	2.7238±0.0788	2.2268±0.0702	0.6485±0.0212
Voting	2.3859±0.0685	1.8730±0.0649	0.7305±0.0122

Abbreviations: CatBoost Regressor (CBR); XGBoost Regressor (XGBR); LightGBM Regressor (LGBMR); Gradient Boosting Regressor (GBR); Random Forest Regressor (RFR); Extra Trees Regressor (ETR); and AdaBoost Regressor (ABR); Voting Regressor (Voting); root mean square error (RMSE); mean absolute error (MAE); R-squared ($R^{2}$).

**Table 14:** Static ensemble regression model results with feature selection and hyperparameter optimization (scale prediction layer).

Model	RMSE	MAE	$R^{2}$
**CBR**	2.3256±0.0515	1.8099±0.0494	0.7439±0.0115
XGBR	2.3773±0.0586	1.8513±0.0593	0.7324±0.0121
LGBMR	2.3825±0.0509	1.8527±0.0456	0.7312±0.0114
GBR	2.3725±0.0498	1.8557±0.0530	0.7335±0.0108
RFR	2.4823±0.0639	1.9236±0.0562	0.7084±0.0106
ETR	2.5648±0.0648	1.9866±0.0604	0.6885±0.0141
ABR	2.6890±0.0673	2.1805±0.0566	0.6576±0.0160
Voting	2.3682±0.0601	1.8738±0.0564	0.7345±0.0122

## Data Availability

The data presented in this study are available in NACDA at https://doi.org/10.3886/ICPSR20541.v10 (accessed on 25 April 2024).

## Supplementary Materials

The following supporting information can be downloaded at: https://www.mdpi.com/article/10.3390/diagnostics14212385/s1, Table S1: Classical ML classifiers model results without feature selection and hyperparamter optimization (detection layer); Table S2: Classical ML classifiers model results with feature selection (detection layer); Table S3: Static ensemble classifiers model results without feature selection and hyperparameter optimization (detection layer); Table S4: Static ensemble classifiers model results with feature selection (detection layer); Table S5: Selecting the best number of classifiers for DES with classical ML classifiers (detection layer); Table S6: Selecting the best number of classifiers for DES with static ensemble ML classifiers (detection layer); Table S7: Selecting the best number of classifiers for DES with a mixed pool of classical and static ensemble classifiers (detection layer); Table S8: Classical ML classifiers model results without feature selection and hyperparamter optimization (severity prediction layer); Table S9: Classical ML classifiers model results with feature selection (severity prediction layer); Table S10: Static ensemble classifiers model results without feature selection and hyperparameter optimization (severity prediction layer); Table S11: Static ensemble classifiers model results with feature selection (severity prediction layer); Table S12: Selecting the best number of classifiers for DES with classical ML classifiers (severity prediction layer); Table S13: Selecting the best number of classifiers for DES with static ensemble ML classifiers (severity prediction layer); Table S14: Selecting the best number of classifiers for DES with a mixed pool of classical and static ensemble classifiers (severity prediction layer); Table S15: Statistics on a selection of numerical features (D: Depression, N: Normal); Listing S1: Hyperparameter optimization search spaces for classical models; Listing S2: Hyperparameter optimization search spaces for static ensemble models; Listing S3: Hyperparameter optimization search spaces for regressor models; Figure S1: Two specific decision rule paths for the detection layer; Figure S2: Two specific decision rule paths for the severity prediction layer; Figure S3: Top 50 most frequent features used in the best model (detection); Figure S4: Top 50 most frequent features used in the best model (severity prediction).
