# Supplementary material for: Explainable Multi-Layer Dynamic Ensemble Framework Optimized for Depression Detection and Severity Assessment
Source: Diagnostics (Basel). 2024 Oct 25;14(21):2385. doi: 10.3390/diagnostics14212385 (PMC11545061; doi:10.3390/diagnostics14212385)
Supplement: Supplementary file 1 [file diagnostics-14-02385-s001.zip › diagnostics-3265372-supplementary.pdf]

(Supplementary materials)

Explainable multi-layer dynamic ensemble framework optimized for depression detection and severity prediction

Dillan Imans[1], Tamer Abuhmed[1], Shaker El-Sappagh[2]
[1] College of Computing and Informatics, Sungkyunkwan University, Suwon, South Korea
[2] Faculty of Computer Science and Engineering, Galala University, Suez, Egypt

Table S1
Classical ML classifiers model results without feature selection and hyperparamter optimization (detection layer)

Model	Accuracy	Precision	Recall	F1-Score	AUC
DT	0.7452 ± 0.0158	0.7456 ± 0.0157	0.7452 ± 0.0158	0.7450 ± 0.0159	0.7452
LR	0.8048 ± 0.0151	0.8053 ± 0.0151	0.8048 ± 0.0151	0.8048 ± 0.0151	0.8847
NB	0.7176 ± 0.0183	0.7234 ± 0.0187	0.7176 ± 0.0183	0.7158 ± 0.0188	0.7852
KN	0.5763 ± 0.0110	0.7217 ± 0.0221	0.5763 ± 0.0110	0.4933 ± 0.0206	0.6735
MLP	0.7806 ± 0.0116	0.7813 ± 0.0114	0.7806 ± 0.0116	0.7805 ± 0.0116	0.8653
**SVC**	**0.8081 ± 0.0184**	**0.8096 ± 0.0187**	**0.8081 ± 0.0184**	**0.8078 ± 0.0184**	**0.8909**

Table S2
Classical ML classifiers model results with feature selection (detection layer)

Model	Accuracy	Precision	Recall	F1-Score	AUC
DT	0.7405 ± 0.0150	0.7412 ± 0.0147	0.7405 ± 0.0150	0.7402 ± 0.0152	0.7405
LR	0.8071 ± 0.0135	0.8075 ± 0.0135	0.8071 ± 0.0135	0.8071 ± 0.0135	0.8889
NB	0.7272 ± 0.0180	0.7309 ± 0.0181	0.7272 ± 0.0180	0.7260 ± 0.0182	0.7987
KN	0.6164 ± 0.0130	0.7309 ± 0.0130	0.6164 ± 0.0130	0.5619 ± 0.0215	0.7059
MLP	0.7899 ± 0.0136	0.7906 ± 0.0135	0.7899 ± 0.0136	0.7898 ± 0.0137	0.8734
**SVC**	**0.8147 ± 0.0125**	**0.8157 ± 0.0129**	**0.8147 ± 0.0125**	**0.8145 ± 0.0125**	**0.8936**

Table S3
Static ensemble classifiers model results without feature selection and hyperparameter optimization (detection layer)

Model	Accuracy	Precision	Recall	F1-Score	AUC
RF	0.8392 ± 0.0098	0.8398 ± 0.0099	0.8392 ± 0.0098	0.8392 ± 0.0098	0.9162
XGB	0.8585 ± 0.0127	0.8587 ± 0.0128	0.8585 ± 0.0127	0.8584 ± 0.0127	0.936
GB	0.8660 ± 0.0123	0.8664 ± 0.0121	0.8660 ± 0.0123	0.8660 ± 0.0123	0.9438
AB	0.8379 ± 0.0122	0.8384 ± 0.0120	0.8379 ± 0.0122	0.8378 ± 0.0123	0.9218
**Cat**	**0.8694 ± 0.0121**	**0.8697 ± 0.0120**	**0.8694 ± 0.0121**	**0.8693 ± 0.0121**	**0.9439**
LGBM	0.8610 ± 0.0138	0.8614 ± 0.0138	0.8610 ± 0.0138	0.8610 ± 0.0138	0.938
Vot	0.8683 ± 0.0119	0.8686 ± 0.0120	0.8683 ± 0.0119	0.8682 ± 0.0120	0.9427

Table S4
Static ensemble classifiers model results with feature selection (detection layer)

Model	Accuracy	Precision	Recall	F1-Score	AUC
RF	0.8280 ± 0.0100	0.8284 ± 0.0101	0.8280 ± 0.0100	0.8279 ± 0.0100	0.9079
XGB	0.8543 ± 0.0152	0.8547 ± 0.0149	0.8543 ± 0.0152	0.8543 ± 0.0152	0.9298
**GB**	**0.8655 ± 0.0134**	**0.8658 ± 0.0134**	**0.8655 ± 0.0134**	**0.8654 ± 0.0134**	**0.9386**
AB	0.8368 ± 0.0160	0.8372 ± 0.0161	0.8368 ± 0.0160	0.8368 ± 0.0160	0.9133
Cat	0.8645 ± 0.0128	0.8649 ± 0.0127	0.8645 ± 0.0128	0.8645 ± 0.0128	0.9385
LGBM	0.8602 ± 0.0115	0.8605 ± 0.0114	0.8602 ± 0.0115	0.8602 ± 0.0115	0.9331
Vot	0.8638 ± 0.0113	0.8642 ± 0.0112	0.8638 ± 0.0113	0.8638 ± 0.0113	0.9377

Table S5
Selecting the best number of classifiers for DES with classical ML classifiers (detection layer)

Experiments	Average Acc	Best Model	Best Model Acc	Worst Model	Worst Model Acc
3 classifiers	0.8255	FIRE-KNORA-E	0.8273	FIRE-METADES	0.8226
4 classifiers	0.8244	FIRE-KNOP	0.8327	FIRE-DESKNN	0.8152
5 classifiers	0.8242	FIRE-KNOP	0.8327	FIRE-DESKNN	0.8184
**6 classifiers**	**0.8229**	**FIRE-KNOP**	**0.8328**	**FIRE-DESKNN**	**0.8142**

Table S6

Selecting the best number of classifiers for DES with static ensemble ML classifiers (detection layer)

Experiments	Average Acc	Best Model	Best Model Acc	Worst Model	Worst Model Acc
3 classifiers	0.8808	FIRE-KNOP	0.8819	FIRE-METADES	0.8797
4 classifiers	0.8794	FIRE-KNOP	0.8817	DESKNN	0.875
**5 classifiers**	**0.8786**	**FIRE-KNOP**	**0.8821**	**DESKNN**	**0.8747**
6 classifiers	0.8693	FIRE-KNOP	0.8819	FIRE-METADES	0.8535

Table S7

Selecting the best number of classifiers for DES with a mixed pool of classical and static ensemble classifiers (detection layer)

Static/Classic	Average Acc	Best Model	Best Model Acc	Worst Model	Worst Model Acc
6/4	0.8679	FIRE-KNOP	0.8755	DESMI	0.8273
4/6	0.8696	KNOP	0.8812	DESMI	0.8278
5/5	0.8704	FIRE-METADES	0.8805	DESMI	0.8235
5/4	0.8708	METADES	0.8808	DESMI	0.827
4/5	0.8696	KNOP	0.8806	DESMI	0.827
4/4	0.8716	FIRE-METADES	0.881	DESMI	0.827
4/3	0.8718	KNOP	0.8812	DESMI	0.8237
3/4	0.8659	KNOP	0.88	DESMI	0.8237
3/3	0.8674	KNOP	0.8798	DESMI	0.8237
3/2	0.8719	FIRE-KNOP	0.8819	DESMI	0.8196
2/3	0.8594	FIRE-KNOP	0.8798	DESMI	0.8237
**3/1**	**0.8747**	**FIRE-KNOP**	**0.8833**	**DESMI**	**0.8187**
1/3	0.8507	KNOP	0.8712	DESMI	0.8242
2/2	0.8686	METADES	0.8812	DESMI	0.8242
2/1	0.8686	METADES	0.8813	DESMI	0.8242
1/2	0.8558	FIRE-KNOP	0.8785	DESMI	0.8242

Table S8
Classical ML classifiers model results without feature selection and hyperparamter optimization (severity prediction layer)

Model	Accuracy	Precision	Recall	F1-Score	AUC
DT	0.6877 ± 0.0196	0.6967 ± 0.0205	0.6877 ± 0.0196	0.6841 ± 0.0202	0.6877
LR	0.7726 ± 0.0229	0.7765 ± 0.0226	0.7726 ± 0.0229	0.7718 ± 0.0231	0.858
NB	0.6710 ± 0.0282	0.6871 ± 0.0208	0.6710 ± 0.0282	0.6628 ± 0.0346	0.7412
KN	0.6010 ± 0.0122	0.7082 ± 0.0345	0.6010 ± 0.0122	0.5430 ± 0.0212	0.7476
MLP	0.7616 ± 0.0214	0.7691 ± 0.0206	0.7616 ± 0.0214	0.7599 ± 0.0219	0.8518
**SVC**	**0.7868 ± 0.0148**	**0.7998 ± 0.0145**	**0.7868 ± 0.0148**	**0.7844 ± 0.0153**	**0.8724**

Table S9
Classical ML classifiers model results with feature selection (severity prediction layer)

Model	Accuracy	Precision	Recall	F1-Score	AUC
DT	0.6894 ± 0.0251	0.6967 ± 0.0249	0.6894 ± 0.0251	0.6864 ± 0.0257	0.6894
LR	0.7861 ± 0.0183	0.7899 ± 0.0176	0.7861 ± 0.0183	0.7854 ± 0.0185	0.8694
NB	0.6929 ± 0.0316	0.7024 ± 0.0270	0.6929 ± 0.0316	0.6887 ± 0.0348	0.7566
KN	0.6855 ± 0.0191	0.7284 ± 0.0247	0.6855 ± 0.0191	0.6701 ± 0.0216	0.7903
MLP	0.7706 ± 0.0154	0.7788 ± 0.0141	0.7706 ± 0.0154	0.7689 ± 0.0160	0.8643
**SVC**	**0.7894 ± 0.0127**	**0.8000 ± 0.0131**	**0.7894 ± 0.0127**	**0.7875 ± 0.0129**	**0.8806**

Table S10
Static ensemble classifiers model results without feature selection and hyperparameter optimization (severity prediction layer)

Model	Accuracy	Precision	Recall	F1-Score	AUC
RF	0.8032 ± 0.0162	0.8284 ± 0.0170	0.8032 ± 0.0162	0.7994 ± 0.0167	0.9105
XGB	0.8229 ± 0.0173	0.8368 ± 0.0143	0.8229 ± 0.0173	0.8210 ± 0.0181	0.9208
GB	0.8223 ± 0.0166	0.8344 ± 0.0143	0.8223 ± 0.0166	0.8206 ± 0.0174	0.9201
AB	0.8090 ± 0.0221	0.8165 ± 0.0209	0.8090 ± 0.0221	0.8078 ± 0.0225	0.9059
Cat	0.8268 ± 0.0206	0.8389 ± 0.0182	0.8268 ± 0.0206	0.8251 ± 0.0213	0.9213
LGBM	0.8216 ± 0.0138	0.8373 ± 0.0124	0.8216 ± 0.0138	0.8195 ± 0.0144	0.9179
**Vot**	**0.8306 ± 0.0189**	**0.8448 ± 0.0149**	**0.8306 ± 0.0189**	**0.8288 ± 0.0200**	**0.9262**

Table S11
Static ensemble classifiers model results with feature selection (severity prediction layer)

Model	Accuracy	Precision	Recall	F1-Score	AUC
RF	0.8077 ± 0.0101	0.8310 ± 0.0098	0.8077 ± 0.0101	0.8043 ± 0.0108	0.9097
XGB	0.8216 ± 0.0163	0.8339 ± 0.0140	0.8216 ± 0.0163	0.8199 ± 0.0169	0.9148
GB	0.8194 ± 0.0199	0.8303 ± 0.0178	0.8194 ± 0.0199	0.8178 ± 0.0207	0.9167
AB	0.8168 ± 0.0254	0.8249 ± 0.0227	0.8168 ± 0.0254	0.8155 ± 0.0262	0.9072
Cat	0.8261 ± 0.0165	0.8379 ± 0.0133	0.8261 ± 0.0165	0.8245 ± 0.0174	0.9192
LGBM	0.8177 ± 0.0140	0.8326 ± 0.0125	0.8177 ± 0.0140	0.8156 ± 0.0147	0.9164
**Vot**	**0.8268 ± 0.0110**	**0.8406 ± 0.0090**	**0.8268 ± 0.0110**	**0.8250 ± 0.0116**	**0.9224**

Table S12
Selecting the best number of classifiers for DES with classical ML classifiers (severity prediction layer)

Experiments	Average Acc	Best Model	Best Model Acc	Worst Model	Worst Model Acc
3 classifiers	0.7832	DESP	0.789	METADES	0.7768
4 classifiers	0.7795	KNORAU	0.7903	FIRE-DESKNN	0.7642
**5 classifiers**	**0.7803**	**KNORAU**	**0.7926**	**FIRE-DESKNN**	**0.7632**
6 classifiers	0.7793	KNORAU	0.7923	DESKNN	0.7568

Table S13
Selecting the best number of classifiers for DES with static ensemble ML classifiers (severity prediction layer)

Experiments	Average Acc	Best Model	Best Model Acc	Worst Model	Worst Model Acc
3 classifiers	0.8289	FIRE-KNOP	0.8319	FIRE-METADES	0.8226
4 classifiers	0.8252	FIRE-KNOP	0.8313	DESKNN	0.8123
**5 classifiers**	**0.8287**	**FIRE-KNOP**	**0.8332**	**DESMI**	**0.8177**
6 classifiers	0.8257	FIRE-KNOP	0.8326	DESKNN	0.8123

Table S14

Selecting the best number of classifiers for DES with a mixed pool of classical and static ensemble classifiers (severity prediction layer)

Static/Classic	Average Acc	Best Model	Best Model Acc	Worst Model	Worst Model Acc
**6/4**	**0.831**	**FIRE-KNOP**	**0.8368**	**DESMI**	**0.8255**
4/6	0.8243	KNOP	0.8332	DESMI	0.8097
5/5	0.8283	KNOP	0.8358	DESMI	0.8165
5/4	0.8317	KNOP	0.8367	KNORAE	0.8281
4/5	0.8256	DESKNN	0.8319	DESP	0.8155
4/4	0.8271	FIRE-KNOP	0.8342	DESP	0.8155
4/3	0.8278	FIRE-KNOP	0.8326	DESMI	0.8123
3/4	0.8222	KNOP	0.8319	DESMI	0.8123
3/3	0.8172	KNOP	0.8313	DESKNN	0.7997
3/2	0.8258	FIRE-KNOP	0.8313	DESMI	0.8123
2/3	0.8095	KNOP	0.8284	FIRE-DESP	0.7987
3/1	0.8251	FIRE-KNOP	0.8329	DESKNN	0.8123
1/3	0.7893	DESMI	0.8123	FIRE-METADES	0.7761
2/2	0.819	FIRE-KNOP	0.829	FIRE-DESP	0.8035
2/1	0.8263	KNORAU	0.8306	FIRE-KNORA-E	0.8181
1/2	0.8049	DESMI	0.8194	FIRE-KNORA-U	0.7923

Table S15

Statistics on a selection of numerical features (D: Depression, N: Normal)

Feature Description	Overall Mean	Mean (D/N)	STD (D/N)	UoM
Age of participant	69.24	69.85/69.10	8.25/7.70	years
Years in current residence	22.73	21.92/22.91	17.82/17.40	years
Average hours of sleep	6.89	6.51/6.98	1.65/1.29	hrs
Number of medications	5.22	6.54/4.92	4.42/3.76	number of
Body mass index	29.1	29.66/28.98	7.79/5.99	bmi
Mean pulse rate	70.72	72.30/70.36	13.27/11.74	bpm
Mean diastolic rate	80	80.24/80.89	12.52/11.73	mmHg
Frequency of sex last year	0.5	0.44/0.57	0.50/0.49	number of
Cotinine result	70.5	80.58/59.44	200/176	ng/mL

Listing S1

Hyperparameter optimization search spaces for classical models.

- **Decision Tree:**
  - Criterion: gini, entropy
  - Max Depth: Integer range [1, 20]
  - Min Samples Split: Integer range [2, 10]
  - Min Samples Leaf: Integer range [1, 10]
- **Logistic Regression:**
  - C: Real range [1e-6, 1e+6], log-uniform prior
  - Solver: lbfgs, liblinear
  - Max Iterations: Integer range [100, 1000]
- **Naive Bayes:**
  - Variance Smoothing: Real range [1e-9, 1e-2], log-uniform prior
- **K-Nearest Neighbors:**
  - Number of Neighbors: Integer range [1, 30]
  - Weights: uniform, distance
  - Metric: euclidean, manhattan, minkowski
- **Multi-layer Perceptron:**
  - Hidden Layer Sizes: Integer range [50, 200]
  - Alpha: Real range [1e-6, 1e-2], log-uniform prior
  - Learning Rate Init: Real range [1e-4, 1e-2], log-uniform prior
- **Support Vector Classification:**
  - C: [0.1, 1, 10, 100, 1000]
  - Gamma: [1, 0.1, 0.01, 0.001, 0.0001]

Listing S2

Hyperparameter optimization search spaces for static ensemble models.

- **Random Forest:**
  - Number of Estimators: [100, 200, 300]
  - Criterion: gini, entropy
  - Max Depth: None, 7, 15
  - Bootstrap: True, False
- **XGBoost:**
  - Number of Estimators: [100, 200, 300]
  - Max Depth: [5, 10]
  - Learning Rate: [0.01, 0.1, 0.2]
  - Gamma: [0, 0.2, 0.4]
- **Gradient Boosting:**
  - Number of Estimators: [100, 200, 300]

- Learning Rate: [0.01, 0.1, 0.2]
- Max Depth: [5, 10]
- Subsample: [0.7, 0.9, 1.0]

- **AdaBoost:**
  - Number of Estimators: [100, 200, 300]
  - Learning Rate: [0.1, 0.5, 1.0]
  - Algorithm: SAMME, SAMME.R
- **CatBoost:**
  - Iterations: [100, 200, 300]
  - Depth: [5, 7, 9]
  - Learning Rate: [0.01, 0.1, 0.2]
- **LightGBM:**
  - Number of Estimators: [100, 200, 300]
  - Number of Leaves: [31, 63, 127]
  - Learning Rate: [0.01, 0.1, 0.2]
  - Subsample: [0.7, 0.9, 1.0]

Listing S3

Hyperparameter optimization search spaces for regressor models.

- **CatBoost Regressor (CBR):**
  - Iterations: Integer range [100, 500]
  - Learning Rate: Real range [0.01, 0.1]
  - Depth: Integer range [3, 10]
- **Gradient Boosting Regressor (GBR):**
  - Number of Estimators: Integer range [50, 300]
  - Learning Rate: Real range [0.01, 0.1]
  - Max Depth: Integer range [3, 10]
- **Random Forest Regressor (RFR):**
  - Number of Estimators: Integer range [50, 300]
  - Max Depth: Integer range [3, 20]
- **XGBoost Regressor (XGBR):**
  - Number of Estimators: Integer range [50, 300]
  - Learning Rate: Real range [0.01, 0.1]
  - Max Depth: Integer range [3, 10]
- **LightGBM Regressor (LGBMR):**
  - Number of Estimators: Integer range [50, 300]
  - Learning Rate: Real range [0.01, 0.1]
  - Number of Leaves: Integer range [20, 50]
- **Extra Trees Regressor (ETR):**

    - Number of Estimators: Integer range [50, 300]
    - Max Depth: Integer range [3, 20]
- **AdaBoost Regressor (ABR):**
    - Number of Estimators: Integer range [50, 300]
    - Learning Rate: Real range [0.01, 1.0]

Figure S1

Two specific decision rule paths for the detection layer

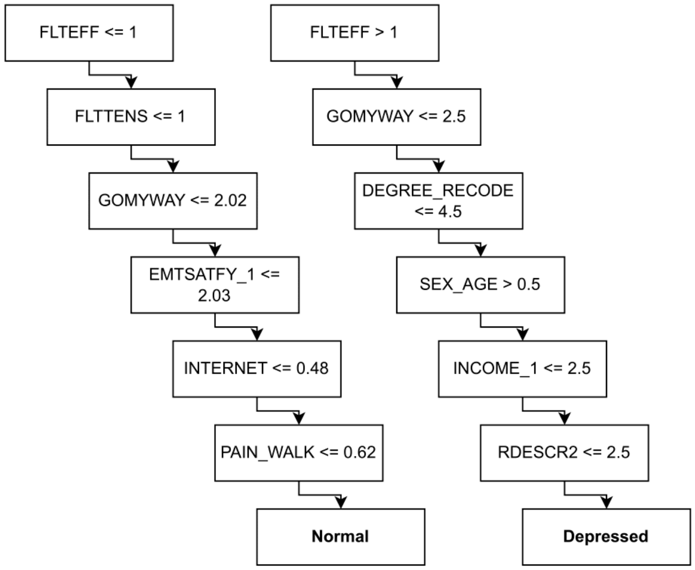

Figure S2

Two specific decision rule paths for the severity prediction layer

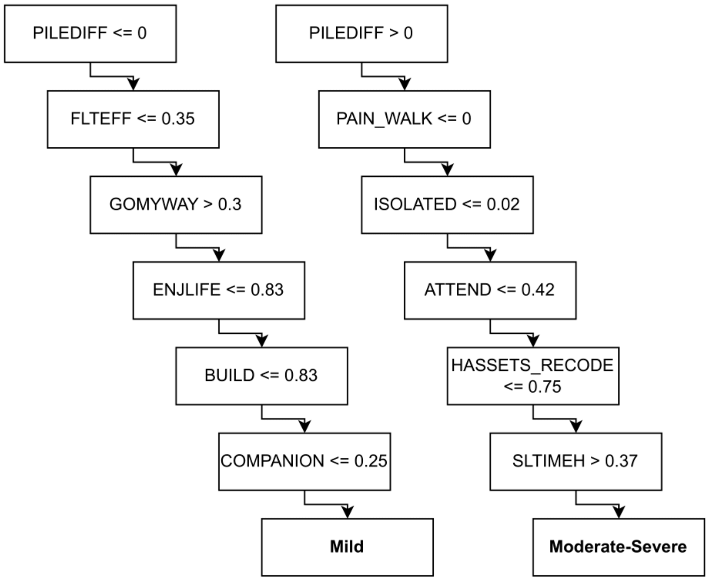

Figure S3

Top 50 most frequent features used in the best model (detection)

Basic Info

BELIEFS: Carry religious beliefs into other dealings in life?

Employment

HEARN_RECODE: Household income (last year) recode
INCOME_2: Household income relative to American families
HSASSETS_RECODE: Total household assets recode

Social Network & Support

RESIDEY: Time lived in neighborhood (years)
RESIDE: Total time lived in neighborhood (derived, in months)
FRSTMARG: Was this your first marriage?
SOCIAL: Frequency of socializing with friends or relatives in past year
COMPANION: UCLA loneliness scale: lack companionship
UNFRIEND: CESD scale: people were unfriendly
ISOLATED: UCLA loneliness scale: feel isolated

Relationship

PLEASURE_1: Loop 1: how physically pleasurable is/was the relationship?

Mental Health

FLTEFF: CESD: felt everything was an effort
GOMYWAY: PSS: things are going my way
ENJLIFE: CESD: enjoyed life
FLTTENS: HADS: felt tense or wound up
WASHAPY: CESD: was happy
BUTRFLY: HADS: butterflies in my stomach
WASLONLY: CESD: felt lonely
FLTSAD: CESD: felt sad
FRIGHT: HADS: something awful about to happen
DISLIKD: CESD: felt people disliked me
PILEDIFF: PSS: difficulties piling up

Physical Health

RESTED: How often do you feel rested in the morning?
PAIN_WALK: Pain walking during the last 12 months?
HLTHVIS: Number of times seen doctor in the last 12 months
CONDITNS_1: Ever had arthritis?

Interviewer's Perspective

STRUCTQ: Type of structure in which respondent lives

Saliva Collection

TESTOSTERONE_1: Result 1 (pg/mL)
TESTOSTERONE_2: Result 2 (pg/mL)
COTININE_1: Result 1 (ng/mL)
COTININE_2: Result 2 (ng/mL)
DHEA_2: Result 2 (pg/mL)
LASTEATH: Hours since last food or drink
LASTEATM: Minutes since last food or drink
PROGESTERONE_1: Result 1 (pg/mL)
PROGESTERONE_2: Result 2 (pg/mL)

Sensory Function

TASTEID_1: Taste identification certainty: sour
TASTEID_2: Taste identification certainty: bitter
TASTEID_4: Taste identification certainty: salty

Anthropometrics

BMI: Body mass index (kg/m^2)
WEIGHT: Weight (lbs)
HEIGHT: Height (inches)
WAISTM: Waist (inches)

Blood Pressure

SYSTOLIC_1: First systolic value (mm Hg)
SYSTOLIC_2: Second systolic value (mm Hg)
DIASTOLIC_1: First diastolic value (mm Hg)
DIASTOLIC_2: Second diastolic value (mm Hg)
PULSE_1: First pulse rate (beats/min)
PULSE_2: Second pulse rate (beats/min)

28 questionnaire features

22 health exam features

Figure S4

Top 50 most frequent features used in the best model (severity prediction)

Mental Health

FLTEFF: Felt everything was an effort
FLTSAD: Felt sad
GOMYWAY: Things are going my way
FLTTENS: Felt tense or wound up
PILEDIFF: Difficulties piling up
FRIGHT: Something awful about to happen
ENJLIFE: Enjoyed life
WASHAPY: Was happy
WASLONLY: Felt lonely
BUTRFLY: Butterflies in my stomach
PANIC: Sudden feeling of panic
LEFTOUT: Feel left out
SLFESTEM: Self-rated self-esteem
HAPPY: Self-rated general happiness
DISLIKD: Felt people disliked me
MNTLHLTH: Self-rated mental health

Employment

WEEKPAY: Worked for pay last week?
JOBSTAT_1: Currently working?
INCOME_1: Household income relative to people you know
DEGREE_RECODE: Education level obtained

Physical Health

SMOKECIG: Do you smoke cigarettes?
RESTED: How often do you feel rested in morning?
EVERSMK: Ever smoke cigarettes regularly?
PAIN_WALK: Pain walking during last 12 months
PHYSHLTH: Self-rated physical health
HLTHVIS: Last 12 months: How many times seen doctor?
DISCUSS_1: Ever discussed sex with doctor?
INOUTBED: Difficulty getting in or out of bed
DRIVEN: Difficulty driving car during day
INSURE_1: Covered by health insurance?
CONDITNS_13: Ever had skin cancer?
PHYSACT: Frequency of physical activity
OTHURINE: Other urinary problems in last 12 months

Social Network & Support

INTERNET: Frequency of internet use
RESIDE: Total time lived in neighborhood (derived, in months)
RESIDEY: Time lived in neighborhood (years)
SOCIAL: Frequency of socializing with friends or relatives in past year

Relationship

SEX_OBLIG: How often had sex because felt obligated?
EMTSATFY_1: How emotionally satisfying is/was the relationship?
RLTHAPPY_1: How happy is/was the relationship?

Sensory Function

TASTEID_2: Taste identification - Bitter

Saliva Collection

TESTOSTERONE_2: Result 2 (pg/mL)
COTININE_2: Result 2 (ng/mL)
COTININE_1: Result 1 (ng/mL)
TESTOSTERONE_1: Result 1 (pg/mL)
PROGESTERONE_2: Result 2 (pg/mL)
DHEA_2: Result 2 (pg/mL)
DHEA_1: Result 1 (pg/mL)
PROGESTERONE_1: Result 1 (pg/mL)
LASTEATM: Minutes since last food or drink

40 questionnaire features

10 health exam features
